# Intensified Bio-Oil Extraction from Microalgae Integrating Renewable and E‑Fuel Production

**DOI:** 10.1021/acsomega.5c12651

**Published:** 2026-06-30

**Authors:** Nícholas Alexandre Berger Bento, Marcelo Silveira Bacelos, Paulo Sérgio da Silva Porto

**Affiliations:** † 28126 Universidade Federal do Espírito Santo, Programa de Pós-graduação em Energia, São Mateus 29932-540, Espírito Santo, Brazil; ‡ 28126 Universidade Federal do Espírito Santo, Departamento de Engenharia e Tecnologia, Programa de Pós-graduação em Energia, São Mateus 29932-540, Espírito Santo, Brazil

## Abstract

The growing demand for alternatives to fossil fuels is driving research into biofuels and e-fuels (i.e., synthetic fuels made from captured CO_2_ and renewable hydrogen via power-to-liquid processes), underscoring their important role in decarbonization and the global energy transition. Microalgae have emerged as a promising resource due to their high lipid productivity (∼80,000 L/ha/year), rapid biomass growth (∼50 g/m^2^/day), and capacity to capture up to 1.83 kg CO_2_/kg biomass. However, challenges related to extraction efficiency, process scalability, and economic viability still limit industrial use. Addressing these issues, this review assesses the recent technological advances in bio-oil extraction from microalgae for renewable and e-fuel production, covering fuel platforms, corefining, and hybrid routes. Research indicates that hydrothermal liquefaction (HTL) as well as ultrasound- and microwave-assisted extraction techniques approach the technical requirements for commercial deployment. Ultrasound- and microwave-assisted extractions achieve lipid yields above 50 wt % (dry basis), while hydrothermal liquefaction enables wet biomass processing with bio-oil yields ranging from 20 to 55 wt %. A comparison with oilseed crops (e.g., soybean: ∼600 L/ha/year; canola: ∼1,200 L/ha/year) reveals that microalgae exhibit superior sustainability and productivity indicators, despite differences in microalgae structure and composition. Based on this review, the adoption of a circular-economy approach is recommended, with biomass pretreatment followed by hybrid extraction, which offers a promising framework to support decarbonization and large-scale e-fuel production.

## Graphical abstract

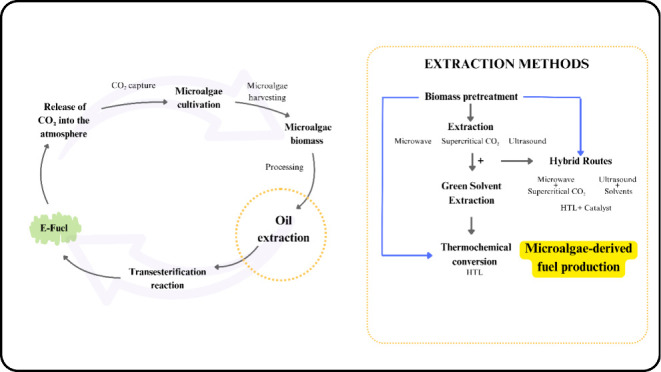

## 1 Introduction

Microalgae stand out as a promising source for producing biofuel and e-fuel due to their high growth rate, efficient CO_2_ fixation, and high lipid production. Saline water microalgae are considered a key element in a circular economy model because they do not compete with agriculture and can use wastewater, transforming it into resources that contribute to sustainable biofuel production. According to global decarbonization policies, bio-oil from biomass thermochemical conversion has the potential to be refined and used in combustion engines, either as a component of diesel or as a direct diesel substitute.

In recent years, various technological methods have been introduced to enhance bio-oil extraction from microalgae. Among these, hydrothermal liquefaction (HTL) is one of the most studied techniques, enabling the direct conversion of wet biomass into biofuels without the need for predrying, thereby greatly reducing energy consumption costs. Lipid extraction, cell disruption techniques, and thermochemical conversion routes are also treated as distinct pathways. Although HTL represents a direct biomass-to-bio-oil conversion process rather than a lipid-extraction method, it is included for comparative purposes as an alternative route for liquid-fuel production from microalgae. Usami et al.^1^ demonstrated that water recycling in HTL can boost bio-oil yields and decrease operating temperatures, making the process more economical and efficient. Similar HTL performance has also been reported in other experimental studies. For example, Arun et al.^2^ produced bio-oil through hydrothermal liquefaction of *Chlorella vulgaris* biomass, achieving a bio-oil yield of approximately 29.4 wt % at 300 °C for 60 min, along with reductions in oxygen and nitrogen content and an increase in calorific value, indicating improved fuel properties. While HTL represents a thermochemical conversion pathway, the primary focus of this review is on lipid extraction technologies and their integration with downstream fuel production systems. Another important advancement involves the development of alternative lipid extraction methods. According to Yang et al.,^3^ the use of ethanol as a solvent at room temperature yielded promising results, eliminating the need for biomass drying. Phusunti and Cheirsilp^4^ showed that the economic potential of microalgae-derived bio-oil production can be increased with integrated protein extraction. The results indicated a lower content of undesirable components, such as acids and nitrogen compounds, and a high bio-oil quality.

One major challenge in microalgae-derived fuel production is the biochemical diversity found among different microalgae species, as this variability affects how efficiently the extraction and conversion processes can be carried out. Kruger et al.^5^ explored different pretreatment methods, including dilute acids and bases, to solubilize carbon and nitrogen, facilitating lipid extraction. The authors demonstrated that the efficiency of these methods varies significantly between different microalgae strains, reinforcing the need to optimize pretreatments for specific species. This variability extends across different taxonomic groups, including marine, halophilic, and extremophilic microalgae, whose distinct biochemical compositions influence process standardization, technology selection, and scalability of extraction and conversion systems. For *Chlorella vulgaris*, the kinetic and thermodynamic study by Usami et al.^1^ showed that the choice of temperature and extraction time directly influences the yield and quality of the bio-oil produced.

With respect to integrated microalgae-to-e-fuel systems, the literature consistently points to two main findings. First, integrating microalgae cultivation with concentrated CO_2_ streams enhances CO_2_ utilization and biomass productivity. This integration produces a lipid-rich, renewable carbon intermediate that improves overall carbon conversion and reduces net life-cycle emissions when cultivation, harvesting, extraction, and upgrading are integrated.^6,7^ Second, high lipid recovery and suitable fuel precursors are more reliably achieved when physical cell disruption and process intensification are coupled with solvent-based and assisted extraction.^8–10^ These stages can also be integrated with upgrading and power-to-liquid synthesis, which are supported by renewable electricity and green hydrogen.^11,12^

As noted, combining microalgal lipid production with renewable energy provides a sustainable approach to integrating carbon capture and synthetic fuel production. Although previous reviews have focused on individual technologies and sustainability, a comprehensive assessment linking bio-oil to e-fuel processing remains limited. Challenges include an integrated framework that combines quantitative performance comparisons with clear conceptual differentiation between lipid extraction and thermochemical bio-oil production and that links them to emerging e-fuel production systems.

These issues hinder the identification of technological bottlenecks and assessment of scalable integration strategies. Therefore, this review presents a system-level framework that integrates physical and chemical lipid extraction methods for microalgae with carbon capture and the generation of synthetic fuels powered by renewable energy.

This review aligns with the integrated algal biorefinery concepts for sustainable biodiesel production proposed by Kumar and Singh^13^ and extends them within a power-to-e-fuel framework. It differs from work by Yue^11^ by incorporating hydrogen production and biomass conversion into a value chain that includes microalgae, CO_2_, H_2_, and e-fuel synthesis. It also distinguishes itself from the work of Pandey et al.^6^ and David et al.^7^ by combining microalgal CO_2_ utilization with downstream power-to-e-fuel system design.

Consequently, this review aims to assess and compare lipid extraction methods by linking them directly to microalgae-derived fuel pathways and examining their limitations, including extraction efficiency, process scalability, and economic feasibility for industrial use. Furthermore, this contribution provides a comprehensive framework that not only assesses extraction methods in terms of yield, energy demand, and cost but also explores their compatibility with integration into renewable fuel systems. This perspective advances the field by identifying strategies to overcome production challenges and recommending environmentally sustainable practices for future fuel development.

The remainder of this review is organized as follows. Lipid extraction technologies are first classified by operating principles and pretreatment requirements, followed by a comparative analysis of extraction yields, energy demands, costs, and environmental impacts. These metrics are then examined in the context of downstream fuel production pathways and system-level integration to assess the scalability and technological relevance of the reviewed approaches.

## 2 Technological and Chemical Advances in Bio-Oil Extraction from Microalgae

Bio-oil extraction from microalgae has attracted increasing attention because of their potential as a sustainable, high-lipid-content biomass source. Over the past decade, significant progress has been made in extraction techniques that target lipid recovery, purity, and sustainability. These methods can be broadly classified into physical, chemical, and thermochemical categories, each with distinct mechanisms, advantages, and limitations. This section critically evaluates the main extraction technologies, highlighting their chemical underpinnings, comparative performances, and scalability.

For conceptual clarity, the discussion follows a hierarchical processing framework consisting of pretreatment/cell disruption, lipid extraction, and thermochemical conversion stages. In this context, lipid extraction technologies are the primary focus of this section, whereas thermochemical conversion routes, such as hydrothermal liquefaction (HTL), are discussed separately and are included primarily for comparative benchmarking within integrated fuel production systems.

### 2.1 Overview and Classification of Extraction Methods

The classification of lipid extraction technologies into physical, chemical, and assisted-energy approaches provides a useful framework for evaluating their performance in relation to downstream fuel production pathways. However, beyond classification, a comparative and integrative analysis is required to identify which techniques effectively drive improvements in the lipid recovery and process efficiency. This classification enables direct comparison of extraction yield, energy requirements, solvent compatibility, and integration potential with upgrading and fuel conversion processes, thereby supporting the identification of technologies with the highest scalability and process integration potential.

Physical approaches, such as ultrasound and microwave-assisted extraction, promote the disruption of cell walls. Chemical techniques, including the use of organic solvents, supercritical CO_2_, and hydrolysis, improve solubility and selectivity. Thermochemical processes, such as hydrothermal liquefaction (HTL), which are treated here as bio-oil production pathways rather than lipid extraction methods, facilitate the direct conversion of biomass into bio-oil fractions. Each methodology offers distinct trade-offs related to selectivity, energy consumption, yield, and environmental compatibility. These trade-offs must be interpreted in the context of operating conditions and biomass characteristics, as performance differences reported in the literature often are not intrinsic to the techniques themselves but rather are related to their experimental configurations.

In this review, the term “lipid extraction” refers to physical or chemical processes that isolate native lipids from microalgae cells without chemical alteration, such as solvent, ultrasound, or supercritical CO_2_ extraction. The term “bio-oil production” refers instead to thermochemical processes such as hydrothermal liquefaction (HTL), where biomass components undergo decomposition and reformation into a complex oil phase. These two concepts are treated distinctly in this review to avoid terminological ambiguity. The term “lipid yield” refers to the mass of extracted lipids expressed on a dry biomass basis unless otherwise specified. This definition is consistently applied throughout the manuscript to avoid redundancy and ensure comparability between sections.

In addition to these approaches, recent studies have explored hybrid extraction combinations to maximize process efficiency. The integration of physicochemical methods, such as the use of ultrasound or microwaves before solvent extraction, has shown significant improvements in lipid release and solvent consumption reduction. Nevertheless, the specific contribution of each technique within hybrid systems is not always clearly distinguished in the literature, which can lead to overgeneralized claims regarding their performance. Moreover, using cosolvents with different polarities enables the selective extraction of desirable compounds, enhancing bio-oil quality. These hybrid strategies present a promising pathway for increasing extraction scalability while minimizing environmental impact.

Organic solvent extraction is a conventional method that uses solvents like hexane, chloroform, or methanol to extract lipids from dry microalgae biomass. In contrast, the green solvent method employs ethanol to extract lipids from wet biomass, eliminating the need for prior drying. In this review, the term “green solvent” refers to solvents characterized by reduced environmental and health impacts, including criteria such as low toxicity, renewability, recyclability, low vapor pressure, and favorable life-cycle environmental performance, recognizing that individual sustainability metrics may vary depending on the assessment framework. Although ethanol is often highlighted as a sustainable alternative, its extraction efficiency for nonpolar lipid fractions may be lower compared to traditional solvents, reinforcing the importance of solvent selection based on the target lipid composition.

Among the process parameters influencing lipid extraction performance, solvent polarity plays a particularly important role, as it directly affects lipid solubility, selectivity, and phase separation efficiency. Therefore, the following studies specifically examine the influence of solvent systems on extraction performance. Although multiple operational parameters influence extraction performance, the following discussion focuses specifically on solvent-related effects with other variables considered only insofar as they influence solvent behavior.

Bio-oil separation from an aqueous solution or biomass typically requires the use of solvents. The yield and properties of the bio-oil depend on the solvent type, quantity, and operational conditions. Vlaskin et al.^14^ found that polar solvents yielded higher amounts of bio-oil from *Arthrospira platensis*, while nonpolar solvents increased residual organic yields. For example, acetonitrile produced nearly double the bio-oil yield compared with dichloromethane and *n*-hexane.

Nonpolar solvents, such as hexane and dichloromethane, primarily extract nonpolar molecules, including lipids and hydrocarbons. This often results in lower bio-oil yields and higher residues, including pigments, long-chain fatty acids, and other hydrocarbons that may have commercial value or be considered byproducts. Yan et al.^15^ examined how solvents influence bio-oil yield and composition. Polar solvents were more effective at extracting ester fractions. These observations reinforce that solvent selection not only affects the yield but also determines the chemical profile of the extracted fractions, which is critical for downstream fuel applications.

Regarding the hydrothermal liquefaction (HTL) process, it converts wet biomass into bio-oil at high temperature (200–370 °C) and pressure (5–25 MPa) without the need of drying. During this process, water acts as both a solvent and a reaction medium, breaking biomass bonds and enabling reactions like depolymerization, deoxygenation, and thermal degradation. The main product, bio-oil, can be refined into liquid fuels like diesel and gasoline. Although HTL is not a lipid extraction method, it is included here for comparative purposes, as it represents an alternative pathway for converting whole biomass into liquid fuels.

Mahima et al.^16^ studied the HTL of *Scenedesmus obliquus* biomass. Pretreated biomass yielded 48 wt % bio-oil compared to 28 wt % from untreated biomass. The bio-oil analysis showed 60% of the compounds were in the diesel and gasoline range, with 58 wt % energy recovery. Low-lipid algae are viable for bio-oil production due to their high yields and adaptability to large-scale cultivation. He et al.^17^ evaluated HTL for two low-lipid algae. *Nannochloropsis* sp. produced higher crude bio-oil yields (39–54 wt %) compared to *Sargassum* sp. (3–9 wt %).

Despite its advantages, HTL faces challenges in maximizing yield and quality due to the high nitrogen and oxygen content in bio-oil.^18,19^ Catalysts can address this issue. Mustapha et al.^20^ applied HTL to nutrient-stressed *Scenedesmus obliquus* with and without a zirconium (Zr)-doped HZSM-5 catalyst. The catalyst improved yield (29 wt %) and reduced carbohydrate content while increasing lipid composition, resulting in a bio-oil resembling crude oil. This observation highlights that HTL performance is strongly influenced by biomass biochemical composition, including lipid, protein, and carbohydrate fractions, rather than feedstock type alone, reinforcing the importance of composition-driven process optimization in thermochemical conversion systems.

Traditional extraction methods like maceration and Soxhlet extraction have significant drawbacks, such as high solvent consumption, long extraction times, and low yields. These limitations drive the search for new techniques, like ultrasound-assisted extraction, which is low-cost compared to conventional methods. Dey and Rathod^21^ studied ultrasound-assisted extraction by optimizing parameters like extraction time, solvent type, biomass-to-solvent ratio, temperature, and acoustic intensity. Under optimal conditions (1.5 g of Spirulina presoaked in methanol for 2 min, 50 mL of *n*-heptane at 30 °C, 167 W/cm^2^ acoustic intensity, 61.5% duty cycle for 8 min, and 0.5 cm probe tip immersion), the extraction yield reached 47%.

Microwave-assisted extraction was evaluated by Mane et al.,^22^ who compared its performance with ultrasound and pretreatment methods. The results showed that ultrasound-assisted extraction achieved three times the yield of microalgae oil compared to microwave-assisted extraction, under specific experimental conditions. However, when broader data sets are considered (Table 1), both techniques present overlapping yield ranges, indicating that such differences are highly dependent on process parameters rather than representing a consistent superiority of ultrasound-assisted extraction.

**Table 1:** Comparative Performance of Microalgal Bio-Oil Extraction Methods^a^

Method	Lipid/Biocrude Yield (wt %)	Energy (MJ kg^–1^ biomass)	Cost (USD kg^–1^ product)	Main Products	Temperature (°C)	Pressure (bar)	Time (min)	References
Ultrasound-assisted	40–55	2–8	1–4	TAGs and FFAs	30–50	∼1	10–30	^8,21,22,26^
Microwave-assisted	35–50	3–7	2–5	TAGs and FFAs	40–100	∼1	5–20	^6,22,26,27^
Hydrothermal liquefaction (HTL)	30–60	8–15	5–12	Alkanes and N/O-compounds	200–370	50–250	20–60	^16,17,20^
Supercritical CO_2_	20–45	8–18	4–10	Neutral lipids	35–60	75–450	30–90	^23,28^
Organic solvent extraction	30–60	10–20	2–6	TAGs and steroids	25–60	∼1	30–90	,^14,15,29^
Acid/base hydrolysis	30–50	6–12	3–7	Mixed lipids and FFAs	60–120	∼1	60–180	^5,25^

a Values represent typical ranges reported or inferred from recent literature. Energy and cost data are indicative and derived from techno-economic or comparative assessments and are not from a single experimental source.

Mechanical pressing shows limited applicability for microalgae due to low efficiency associated with their small cell size and rigid cell walls. The process has significant drawbacks; few studies focus on pressing lipids from microalgae due to the low efficiency. Valdovinos-García et al.^23^ showed that a single method often fails to achieve high oil extraction efficiency. Pretreatments, such as ultrasound or microwave-assisted treatment, are commonly recommended to enhance lipid release from microalgae. Combining mechanical and chemical methods can boost lipid yield by up to 75% by weight, making extraction more efficient and productive. Mechanical pressing is generally considered to have limited applicability for microalgal biomass due to the small cell size and rigid cell walls of many species, which significantly reduce extraction efficiency. For this reason, this method is not included in Table 1, which focuses on the extraction technologies most relevant to microalgae processing.

According to Tzima et al.,^25^ supercritical CO_2_ extraction enables lipid recovery without toxic solvent residues, although it requires high-pressure equipment, which increases operational costs. Acid/base hydrolysis improves lipid recovery by facilitating cell disruption, although it presents operational limitations such as corrosion, salt formation, and downstream treatment requirements. Acid hydrolysis uses acids, such as sulfuric acid, to degrade microalgae cell walls, enabling lipid extraction with solvents, particularly in wet biomass. Alkaline hydrolysis uses bases such as sodium hydroxide to degrade cell structures, thereby enhancing the lipid release and extraction efficiency. These chemical treatments improve lipid recovery and increase the overall yield in biorefinery systems.^25^

### 2.2 Comparative Efficiency of Extraction Methods

A direct comparison of performance indicators such as lipid yield, energy consumption, operating conditions, and product composition is essential for selecting an appropriate technique. Table 1 summarizes the main parameters for the most representative methods and serves as the basis for the comparative analysis discussed below. However, these values should be interpreted with caution as they are derived from different studies with varying biomass compositions, operating conditions, and experimental setups, which may limit direct comparability.

A comparative analysis of the methods in Table 1 reveals distinct profiles in terms of performance, operational demands, and applicability. Thermochemical processes such as hydrothermal liquefaction (HTL) achieve relatively high yields (30–60%) and can process wet biomass, offering a logistical advantage. However, this comes with trade-offs: high-pressure and high-temperature requirements increase operational costs and complexity. Thus, while HTL demonstrates competitive yields, its advantages are strongly dependent on process integration and energy recovery strategies. Although HTL is not strictly a lipid extraction method, it is included in Table 1 as a benchmark conversion pathway for comparison with extraction-based technologies.

Among physical methods, ultrasound-assisted extraction consistently yields 40–55 wt % under mild conditions (30–50 °C), typically with solvent-to-biomass ratios below 5:1, thereby reducing solvent consumption and making it suitable for integration with green solvents in biorefineries. Microwave-assisted methods also provide competitive yields (35–50 wt %) but may cause localized overheating and degradation of sensitive compounds. Although some studies report significantly higher yields for ultrasound-assisted extraction compared to microwave-assisted methods, the ranges presented in Table 1 indicate that, under optimized conditions, both techniques can achieve comparable performances. This suggests that reported differences are often influenced by experimental parameters rather than an intrinsic superiority of one method over the other.

Supercritical CO_2_ extraction exhibits the highest selectivity for neutral lipids and typically produces extracts with purities above 90%, depending on operating pressure and the use of cosolvents; however, the yield (20–45 wt %) is generally lower than that with HTL or ultrasound. Its requirement for high-pressure equipment (up to 450 bar) limits accessibility to industrial applications unless offset by added-value coproducts. Therefore, its applicability is often linked to high-value product recovery rather than bulk fuel production.

Solvent-based extractions, while yielding 30–60 wt %, remain reliant on toxic solvents and dry biomass, thereby increasing energy input. Performance is highly dependent on solvent polarity and target compound class. For instance, hexane selectively extracts TAGs, while ethanol extracts a broader lipid range. This selectivity implies that higher yields do not necessarily correspond to higher-quality fuel precursors, reinforcing the need to evaluate both quantity and composition.

Acid/base hydrolysis serves as an effective pretreatment step, especially for recalcitrant strains, achieving moderate yields (30–50 wt %); however, it often results in degraded proteins and necessitates downstream neutralization. Consequently, its role is better defined as a complementary pretreatment rather than a standalone extraction method.

Overall, the comparison demonstrates that no single method simultaneously optimizes the yield, energy efficiency, cost, and environmental performance. Instead, the relative advantage of each technique depends on the target product, biomass characteristics, and level of process integration. This explains the discrepancies frequently observed across studies and highlights the importance of standardized comparison criteria.

These findings also indicate that hybrid configurations, which combine complementary mechanisms such as cell disruption and selective solvation, are more likely to achieve balanced performance across multiple criteria than individual techniques alone.^21,22,23^ In addition to yield, differences in product composition (e.g., TAG-rich extracts versus nitrogen-containing bio-oils from HTL) further influence downstream upgrading requirements and the overall process viability.

### 2.3 Mechanistic and Chemical Overview

The underlying chemistry of each method defines its selectivity and extraction efficiency, as shown by indicators in Table 2, where the relative yield refers to the approximate recovery efficiency of lipid classes in relation to the total extractable lipids, based on representative values reported in the literature. Solvent-based extractions work by solubilizing lipids based on their polarity. For instance, nonpolar solvents (e.g., hexane) extract triacylglycerols (TAGs), thereby yielding higher total lipid yields from microalgal and animal biomass^30–32^ and thus resulting in extracts with a higher proportion of saturated fatty acids.^30^ In comparison, polar solvents (e.g., ethanol, methanol) are effective for phospholipids and glycolipids.^31,33–35^ This polarity-driven selectivity impacts both the yield and the product profile. Polar solvents are less efficient for TAGs and other nonpolar lipids, with recoveries of less than 5% in some cases,^31^ but can extract fractions rich in oxygenated groups and carbohydrate residues.^33,34^

**Table 2:** Chemical Indicators for Extraction Solvents

Solvent	Polarity	Main Lipids Extracted	Relative Yield (Lipid Recovery Relative to Total Extractable Lipids)	References
Hexane/chloroform	Nonpolar	TAGs, cholesterol esters	High (∼70–90%)	^30–32^
Methanol/ethanol	Polar	Phospholipids, glycolipids, lysophospholipids	Medium/low (∼40–70%)	^31,33–35^
Solvent mixtures (MTBE/MeOH/IPA)	Polar/nonpolar	Broad spectrum (polar and nonpolar)	High	^36,37^

Combinations such as MTBE/methanol/isopropanol can efficiently extract both polar and nonpolar lipids, simplifying the process and increasing the yield.^36,37^ In addition, solvents that change polarity with CO_2_ allow for the selective extraction and easy separation of lipids, resulting in high yield and purity.^38,39^ Another strategy could be the sequential use of solvents of increasing polarity, allowing the fractionation of different classes of lipids and associated compounds.^33^ The choice of solvent determines not only the total yield but also the fatty acid profile and composition of the extracts, impacting properties such as oxidative stability and quality for applications such as biodiesel or functional foods.^30,35,36^

Hydrothermal liquefaction (HTL) promotes hydrolysis, deoxygenation, and depolymerization reactions in subcritical water. Proteins and carbohydrates may be converted into oil-phase compounds, increasing the yield. However, they also introduce nitrogen- and oxygen-rich species, as illustrated in Figure 1, including pyrroles and phenolics, that require further upgrading.

**Figure 1:**
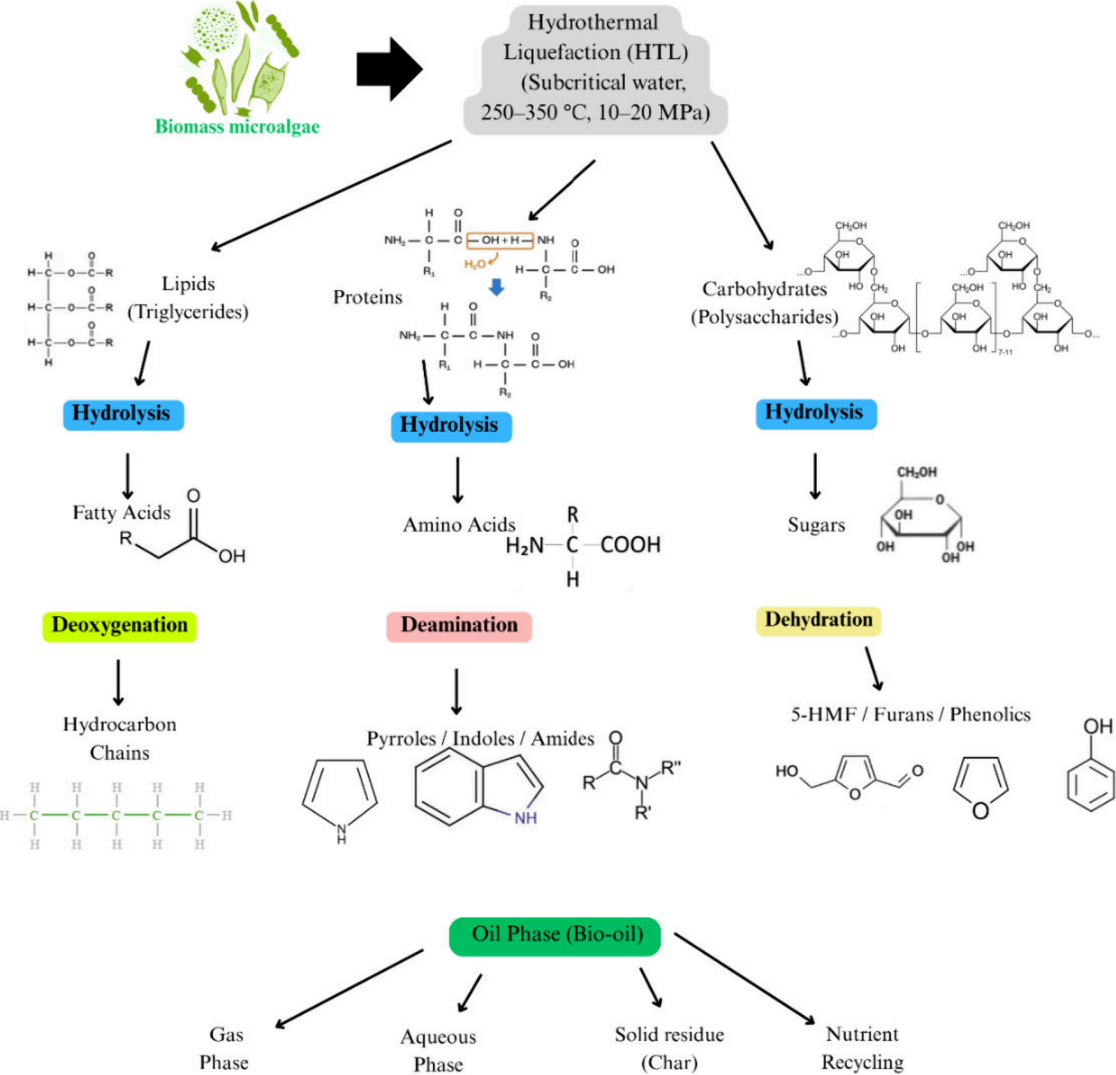
Mechanism involved in the hydrothermal liquefaction process.

In the hydrolysis and depolymerization mechanism, subcritical water promotes the breakdown of polymers, including cellulose, proteins, and plastics, into smaller molecules. For cellulose, the amorphous regions are converted more rapidly than the crystalline ones, producing glucose and 5-hydroxy­methyl­furfural (5-HMF) as the main products.^40,41^ In the deoxygenation mechanism, oxygen is removed from biomass-derived compounds, improving the quality of the resulting bio-oil. The extent of deoxygenation depends on the raw material and the process conditions.^42–44^ Therefore, proteins and carbohydrates in the feedstock can be converted into oil-phase compounds, increasing the yield but also introducing nitrogenous species as well as amides, heterocycles, and oxygenated compounds such as phenolics and furans.^43,44^

The main advantage of ultrasound-assisted extraction lies in its ability to disrupt cell walls through cavitation, which is associated with the key parameters depicted in Table 3. The ultrasonic waves create microscopic bubbles that collapse violently, leading to mechanical rupture of the cell walls and increasing solvent penetration and mass transfer.^26,45–47^ This results in faster, more efficient extraction with reduced solvent and energy consumption while preserving the integrity of heat-sensitive compounds. Additional mechanisms include fragmentation, erosion, capillarity, and sonoporation, all of which contribute to improved extraction efficiency.^45,46,48^ These cavitation-induced effects impact extraction efficiency, lipid class selectivity, and solvent penetration, which in turn influence downstream fuel quality and energy use in the process.

**Table 3:** Key Processing Factors

Parameter	Impact on Extraction	References
Ultrasonic power	Higher power increases disruption and yield, but excessive power may degrade compounds	^26,45–48^
Frequency	Affects cavitation intensity and efficiency	
Temperature	Moderate temperatures optimize yield and protect sensitive compounds	
Extraction time	Most extraction occurs in the first few minutes	
Solvent/sample ratio	Influences mass transfer and extraction efficiency	

Microwave treatment significantly increases biomass and lipid content in microalgae, with reported improvements in total lipid production and enhanced exopolysaccharide yields.^49^ As illustrated in Table 4, this process enhances cell wall disruption, increases the release of intracellular contents and improves the efficiency of downstream applications such as lipid extraction, biodiesel production, and biogas generation.^50^ Direct extraction of wet microalgae results in greater lipid extraction and biodiesel yields compared to those of conventional methods, achieving up to 77.5% cell wall disruption and 1.3 to 1.5 times higher biodiesel yields.^51,52^ Such pretreatment enhances biomass solubilization, leading to increased biogas and methane yields during anaerobic digestion; however, energy input must be taken into account for process efficiency.^51,53^

**Table 4:** Process Enhancement Using Microwaves

Application	Effect of Microwave Irradiation	References
Lipid extraction	Higher yield, easier extraction	^49–53^
Biodiesel production	Increased yield, faster conversion	
Biogas production	Enhanced solubilization, higher methane yield	
Biomass/lipid content	Significant increases in both	

Supercritical CO_2_ interacts preferentially with neutral and nonpolar lipids; however, the addition of cosolvents, such as ethanol, expands the extraction to include polar lipids, as shown in Table 5. This pretreatment is widely used for lipid removal due to its surface layer containing nonpolar and neutral compounds, which interact primarily through van der Waals and dipole forces. It is especially effective for solubilizing neutral lipids like triglycerides due to these interactions.^54–56^

**Table 5:** CO_2_/Cosolvent Interaction

Solvent Type	Extracted Compounds	References
CO_2_ pure	Neutral/nonpolar (triglycerides)	^54–56^
CO_2_ + ethanol	Polar and nonpolar (phospholipids and glycolipids)	
CO_2_ + vegetable oils	Polar, carotenoids (polyunsaturated fatty acids)	

The addition of cosolvents, such as ethanol, enhances the extraction capacity by enabling the extraction of more polar lipids, including phospholipids and glycolipids, which are poorly soluble in pure CO_2_.^56^ This adjustment makes the process more versatile and efficient. Cosolvents also increase the yield of bioactive compounds such as polyunsaturated fatty acids and carotenoids.

Representative hybrid configurations include ultrasound–solvent extraction, microwave-assisted solvent systems, and supercritical CO_2_ extraction combined with polar cosolvents, which have been reported to enhance lipid recovery compared with standalone extraction processes; representative performance data for these systems are summarized in later sections and corresponding tables.

## 3 Sustainability and Processing Scale-Up Assessment

The increasing worldwide demand for renewable fuels has greatly expanded the biofuel market, particularly in the context of decarbonization targets and the energy diversification matrix. In this context, biodiesel production will continue to be driven by both demand from the transportation sector and public policies promoting domestic consumption in several countries. Projections by the OECD/FAO^57^ indicate that biofuel use is expected to expand substantially over the next decade, with global biodiesel production reaching up to 66.9 billion liters by 2032, while remaining heavily reliant on traditional raw materials and processes, such as methanol transesterification. For clarity, in this review “energy efficiency” is defined as the ratio between the energy content of the recovered lipid or produced fuel and the total process energy input required for cultivation, harvesting, drying, extraction, and upgrading steps, depending on system boundaries. “Process cost” refers to the economic requirements of the technology, including operational expenditures (OPEX), such as energy, solvents, labor, and maintenance, as well as capital expenditures (CAPEX), including equipment and infrastructure investments. These definitions are adopted throughout this section to enable consistent comparison among technologies.

In biodiesel production, alcohol is an essential reagent in the transesterification reaction of vegetable oil or fat. Industrially, methanol is widely preferred due to its high reactivity with triglycerides, wide availability, and low cost.^58^ It is estimated that for every million liters of biodiesel produced, up to 300 L of methanol is consumed.^59,60^ Figure 2 graphically outlines global biodiesel production in recent years and an estimate of the consumption of methanol used in the process.

**Figure 2:**
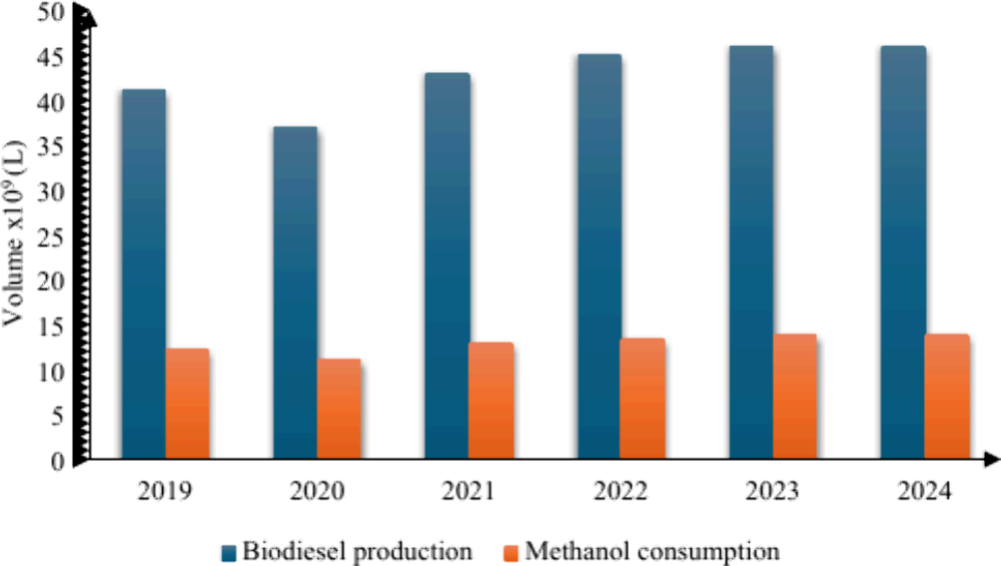
Estimation of methanol consumption in biodiesel production.

As illustrated in Figure 2, the continuous rise in biodiesel production has been accompanied by a proportional increase in methanol consumption, raising important questions about the environmental and economic impacts of solvent use in the biofuel industry. In light of this, growing attention has been directed toward alternative processing strategies aimed at minimizing solvent dependency and improving the sustainability of biodiesel production systems.

Reported life-cycle assessment (LCA) and energy return on investment (EROI) values in the literature depend strongly on the system boundaries adopted (e.g., cradle-to-gate versus cradle-to-wheel) and on assumptions regarding coproduct allocation, waste heat utilization, and energy recovery strategies.

In life-cycle assessment (LCA), as defined in work by Hauschild et al.^61^ and Mazuchi,^62^ “cradle-to-gate” refers to a system boundary that includes raw material extraction, processing, and product manufacturing up to the factory gate, excluding distribution, use, and end-of-life stages. Unless otherwise specified, the cited values herein refer to cradle-to-gate assessments. Recent LCA and TEA (techno-economic analysis) studies have provided updated insights into the environmental performance of microalgal biofuel production systems, comparing lipid extraction and thermochemical conversion routes. Reported EROI values range from 0.3 to 2.1 for extraction-based processes and up to 3.5 for hydrothermal liquefaction (HTL) with energy recovery.^62,63^ GHG emissions vary widely depending on the energy source for cultivation and drying, from 25 to 120 g CO_2_-eq MJ^–1^ of fuel produced.^6^ The water footprint remains a critical constraint, but integration of wastewater or nutrient recycling can reduce freshwater demand by 30–50%.^26^ Higher EROI values reported for HTL pathways often assume partial energy recovery from the aqueous phase, the utilization of residual heat streams, and the allocation of coproduct credits, which can significantly influence the resulting performance indicators.

These findings indicate that system integration and coproduct valorization are crucial to improve overall sustainability and that future developments should focus on coupling renewable hydrogen and CO_2_ reuse to achieve carbon-neutral or even carbon-negative pathways. For example, bio-oil upgrading through hydrogenation using renewable hydrogen, combined with CO_2_ capture and utilization in power-to-liquid processes, can enable integrated pathways for producing low-carbon synthetic fuels. Similar sustainability trends have been reported in recent life-cycle and process integration studies, reinforcing that coupling extraction, conversion, and resource recovery strategies is essential for improving the environmental performance of microalgae-based fuel systems.^62,63^

From a sustainability perspective, methodologies that diminish solvent usage, facilitate the processing of wet biomass, or employ benign solvents (for instance, ethanol, CO_2_) are considered preferable. The sustainability performance discussed here is evaluated with reference to the extraction technologies previously described in Section 2 and summarized in Tables 1 and 2, enabling a direct comparison of energy demand, solvent requirements, and environmental impacts across the reviewed methods. Hydrothermal liquefaction (HTL) and ultrasound-assisted techniques permit extraction without the necessity of prior drying, thereby reducing energy requirements. Nevertheless, HTL demands substantial energy input for heating and pressurization, which may counterbalance its advantages unless process integration is implemented. Supercritical CO_2_ extraction is notable for its purity and environmental sustainability; however, the elevated capital costs and technical intricacies hinder its scalability.

Organic solvent extraction, using substances like hexane and methanol, is effective for extracting lipids from dried microalgae biomass. However, it requires high energy and involves toxic solvents, generating harmful waste. Ethanol, a greener alternative, is less toxic and more sustainable. Although ethanol may exhibit lower selectivity for nonpolar lipid fractions compared with traditional nonpolar solvents, its reduced toxicity, renewability, and lower environmental impact make it a viable alternative for sustainable extraction processes, reflecting the solvent polarity–selectivity relationships discussed in Section 2.

Assisted-energy extraction technologies, including ultrasound- and microwave-assisted methods, are characterized by shorter processing times and reduced solvent consumption compared with conventional extraction approaches, contributing to an improved sustainability performance.

Supercritical CO_2_ extraction uses carbon dioxide in a supercritical state to extract lipids without toxic residues. This environmentally safe method offers high selectivity for lipids but faces challenges due to its high equipment and operating costs.

Key performance indicators for evaluating microalgae lipid extraction processes include the lipid yield, energy efficiency, process cost, bio-oil composition, extraction time, and environmental impact. These parameters are strongly interdependent and must be analyzed simultaneously as improvements in one aspect may lead to trade-offs in others, such as increased energy consumption or operational costs. Therefore, a comprehensive assessment framework is essential to identifying optimal extraction strategies that balance technical performance, economic feasibility, and environmental sustainability.

## 4 Technological and Methodological Solutions

The technological strategies discussed in this section address these challenges through complementary solution categories. Hybrid extraction systems primarily aim to enhance selectivity and reduce coextraction of undesired components. Process intensification approaches focus on lowering energy demand, reducing equipment size, and improving operational efficiency. Solvent and catalyst innovation strategies aim to improve extraction selectivity, reduce solvent consumption, and minimize environmental impacts. This structured classification facilitates the evaluation of the scalability and industrial applicability of proposed technological solutions.

For clarity, the technological solutions discussed in this section are categorized into three groups: (i) hybrid extraction systems, which combine multiple extraction or pretreatment techniques to improve lipid recovery; (ii) emerging extraction concepts, referring to novel process configurations or extraction mechanisms under development; and (iii) auxiliary separation technologies, such as membrane-based systems and microfluidic platforms, which are primarily designed to enhance downstream separation and purification efficiency rather than the extraction step itself.

In order to address the existing challenges associated with microalgal bio-oil extraction and to progress toward commercial viability, it is essential to investigate synergistic, sustainable, and scalable solutions. The primary challenges include the following:

- Low selectivity for specific lipid classes (e.g., neutral versus polar lipids).
- Coextraction of undesirable compounds (e.g., pigments, proteins, chlorophyll).
- Elevated energy or solvent demands.
- Equipment costs and operational safety concerns related to supercritical systems.

As a result, hybrid extraction systems (e.g., ultrasound + ethanol, microwave + supercritical CO_2_) have been proposed. Previous experimental and modeling studies have shown that hybrid extraction configurations can enhance lipid recovery and solvent utilization compared with standalone processes, supporting their growing adoption as a key pathway toward scalable microalgae biorefinery implementation.^28,64–66^ These systems combine physical disruption with selective solvation, enhancing yield while minimizing energy input and environmental impact. Cosolvent systems (e.g., ethanol/hexane azeotropes) have demonstrated improved extraction profiles, efficiently capturing both polar and nonpolar lipids. Process optimization, including kinetic modeling, solvent recycling, and reactor design, represents active research areas aimed at making these methods commercially viable. While no single method is universally superior, the key to advancing bio-oil extraction toward industrial implementation lies in combining complementary techniques and optimizing parameters for specific microalgae strains and products.

To address the challenges of efficiency and sustainability in bio-oil extraction from microalgae, researchers have explored a range of technological and methodological advances to optimize the process. Recent innovations focus not only on improving bio-oil yield and quality but also on minimizing environmental impact and reducing operational costs. These advancements include integrating traditional extraction with emerging technologies and developing hybrid processes that combine multiple methods to enhance the overall process efficiency.

### 4.1 Integration of Hybrid Extraction Systems

Hybrid systems for microalgae lipid extraction have gained attention by integrating traditional extraction methods with innovative techniques to maximize the bio-oil yield and purity. These systems effectively address the limitations of individual methods, enhancing the process selectivity, energy efficiency, and sustainability. Hybrid extraction systems combine two or more extraction or pretreatment techniques in order to exploit complementary mechanisms such as cell disruption and selective solvation, resulting in improved overall process performance compared with that of standalone approaches.

Patil et al.^29^ investigated a hybrid approach combining supercritical CO_2_ extraction with cosolvents and microwave pretreatment. This method significantly improved the lipid extraction efficiency by increasing cell permeability and facilitating lipid mass transfer to the solvent. The use of mixed-polarity cosolvents, such as an azeotropic mixture of hexane and ethanol, proved to be effective in extracting both polar and neutral lipids, optimizing the total yield and quality of the bio-oil. This demonstrates that hybridization enables the simultaneous enhancement of extraction yield and selectivity, which are often competing objectives in single-method systems.

Microwave pretreatment, followed by supercritical CO_2_ extraction with cosolvents, disrupts cell walls, increases the contact surface, and reduces energy consumption. Despite these advantages, large-scale implementation presents challenges, including high equipment costs and the need for the precise control of pressure and temperature. Further research is recommended to assess the economic viability and environmental impacts of these hybrid systems.^28^ Thus, while hybrid systems show strong performance on the laboratory scale, their scalability remains a key limitation.

Additional hybrid configurations reported in the literature include ultrasound–solvent systems and combined physicochemical pretreatments, which consistently demonstrate improved lipid recovery compared with isolated techniques. A study demonstrated the use of a hybrid liquid biphasic system (HLBS) for lipid extraction from microalgae, achieving a recovery of 92.84% and an 8.3-fold enhancement in cell disruption in the organic solvent phase.^64^ In turn, an integrated extraction configuration using switchable solvents in controlled microscale systems resulted in almost 2 times higher total extracted lipids compared to those of traditional methods.^65^

These results indicate that hybrid systems are particularly effective when they combine mechanisms that enhance both mass transfer and solvent selectivity. However, despite these promising results, most studies remain limited to laboratory-scale demonstrations, and there is still a lack of long-term operational data and pilot-scale validation.

It is important to note that some technologies, such as membrane-based separation, pulsed electric fields, and microfluidic systems, are more appropriately classified as process intensification or auxiliary separation strategies rather than hybrid extraction systems. Therefore, their role should be interpreted as complementary to hybrid extraction rather than intrinsic to it.

Overall, hybrid extraction systems represent a key pathway for improving lipid recovery efficiency while reducing solvent consumption and energy demand. However, their successful industrial implementation will depend on the ability to integrate these systems into scalable and economically viable process configurations, particularly within multiproduct microalgae biorefinery.

Compared with standalone extraction methods, hybrid systems consistently demonstrate improved performance by simultaneously enhancing mass transfer and solvent selectivity. However, their effectiveness is highly dependent on the compatibility between the combined techniques and process conditions, which explains the variability observed across different studies.

### 4.2 Process Intensification Strategies

Process intensification strategies focus on improving the efficiency and compactness of the extraction processes. They aim to reduce energy consumption, equipment size, and process steps by integrating operations or applying advanced reactor configurations, such as microreactors, pulsed electric fields, or high-shear systems.^11,67^ Unlike hybrid systems, process intensification often involves rethinking the process design and operation, targeting a higher throughput and lower environmental impact. Process intensification differs from hybrid extraction approaches in how it focuses on the spatial and operational integration of process steps, continuous-flow operation, equipment miniaturization, and multifunctional reactor designs, rather than simply combining multiple extraction techniques.^68^ Within this context, process intensification can be more effectively understood through key underlying principles, including process integration, continuous operation, and multifunctionality, rather than as a collection of individual technologies.

Several studies have highlighted the potential of continuous and integrated systems, such as flow-through extractors coupled with assisted-energy sources (ultrasound or microwave), microreactors, membrane-assisted extraction systems, and multistage separation configurations. These technologies have demonstrated promising performance at the laboratory scale, not only improving extraction yields but also reducing the processing time and environmental burden. These configurations exemplify process integration by combining extraction and separation steps into a single operational framework, thereby minimizing intermediate handling and energy losses.

Integrating the extraction step directly into cultivation units (such as in photobioreactor recirculation systems) is another relevant process intensification strategy, as it eliminates the need for biomass harvesting and drying, thereby minimizing biomass losses and operational costs.^69,70^ This approach reflects the principle of process integration at the system level, where upstream and downstream operations are coupled to enhance the overall efficiency. To enhance clarity, individual technologies such as ultrasound, microwaves, and supercritical CO_2_ should be interpreted within these intensification principles rather than as standalone solutions.

To enhance energy efficiency and reduce costs in bio-oil extraction from microalgae, researchers have explored several emerging technologies including microwave- and ultrasound-assisted extraction, supercritical CO_2_, advanced pyrolysis, and enzymatic hydrolysis. Each method offers unique advantages and contributes to developing a more efficient and sustainable extraction process.^71,72^ However, their contribution to process intensification depends on how they are integrated into continuous or multifunctional systems rather than on their isolated performances.

Microwave-assisted extraction and ultrasound are particularly effective for lipid extraction, enabling rapid and efficient cell disruption. Similarly, ultrasound can be applied as an inline pretreatment unit to enhance mass transfer prior to extraction, enabling integrated extraction–separation systems. Ultrasound-based systems enhance cell disruption through cavitation mechanisms (as previously discussed in Section 2.3). Microwaves, on the other hand, uniformly heat the biomass, significantly reducing both time and energy consumption compared to traditional methods.^27,73^ When applied in continuous-flow configurations or coupled with solvent extraction units, these technologies contribute directly to process intensification by reducing residence time and improving mass transfer efficiency.

Supercritical CO_2_ extraction uses carbon dioxide in its supercritical state as a solvent, offering the dual benefits of being nontoxic and leaving no chemical residues in the bio-oil. Despite its high initial costs, this method is highly efficient and sustainable. Recent research has focused on optimizing operational parameters to reduce costs and maximize lipid yields, positioning supercritical CO_2_ as a key technology for microalgae biorefineries.^74–76^ From a process intensification perspective, its main advantage lies in the integration of extraction and separation in a single step, eliminating the need for solvent recovery stages.

Enzymatic hydrolysis offers a selective method for breaking down microalgae cells, efficiently releasing lipids with a minimal use of aggressive chemicals. This technique preserves lipid integrity, improving bio-oil quality and aligning with sustainable processing methods. However, challenges remain in scaling this approach economically for industrial applications.^77^ Its contribution to process intensification is associated with mild operating conditions and reduced energy input, although limitations in the reaction time and enzyme cost still constrain its large-scale implementation.

These innovative technologies represent significant advancements in the field of process intensification applied to bio-oil extraction, reflecting a growing trend toward replacing conventional extraction routes with integrated and sustainable alternatives. By integrating advanced methods and optimizing processes, these approaches aim to reduce costs, minimize the environmental impact, and support the broader adoption of bio-oil as a renewable energy resource. Overall, the effectiveness of process intensification strategies depends less on individual technologies and more on their integration into coherent process designs that maximize the energy efficiency, minimize unit operations, and enable continuous processing.

These process intensification approaches are consistent with previously reported benchmarks demonstrating that integrated extraction and reactor configurations can reduce processing time and improve overall extraction efficiency compared with conventional batch systems.^73,77–79^

### 4.3 Solvent and Catalyst Innovation

Recent advancements in solvent engineering have opened new pathways for the selective and environmentally responsible extraction of lipids. These innovations aim to improve the extraction efficiency, reduce the environmental impact, and enhance solvent selectivity.

Green solvents, including organic-based solvents, ionic liquids, and supercritical CO_2_, have been investigated as alternatives to traditional fossil-derived solvents. These solvents are eco-friendly and have shown potential for use in lipid extraction processes. For instance, cyclopentyl methyl ether and 2-methyl­tetra­hydro­furan have been utilized for lipid extraction, while imidazolium-based ionic liquids have also proven to be effective.^80^

Supercritical fluid extraction, especially that using CO_2_, has gained popularity because of its ability to extract nutraceutical compounds from biological materials. It is recognized for producing higher levels of antioxidants in extracted lipids compared with conventional methods. Moreover, supercritical CO_2_ extraction is well-established for generating high-value seed oils and other specialty lipids, with ongoing research aimed at integrating enzymes and new gases to enhance the process.^81^

Accelerated solvent extraction (ASE) has been adapted for the selective separation of lipid fractions on the basis of saturation and polarity. This method uses a combination of Ag^+^-impregnated silica gel and solvents with variable eluotropic strength to achieve high recovery rates and the efficient separation of lipid classes.^82^ Similarly, a systematic methodology combining molecular simulation, data classification, and process simulation has identified limonene and ethyl *tert*-butyl ether as promising alternatives to hexane for extracting lipids from algal biomass, due to their greater selectivity and nonhazardous nature.^83,84^

Table 6 summarizes the advantages and disadvantages of using solvents and other techniques. Despite these advances, challenges remain in optimizing the economic viability and environmental sustainability of these processes. For example, although green solvents such as limonene and ethyl *tert*-butyl ether offer greater selectivity, they also entail higher energy requirements and operating expense costs.^84^

**Table 6:** Advantages and Challenges for Solvent/Technique Use

Solvent/Technique	Advantages	Challenges	References
Green solvents	Environmentally friendly, effective in lipid extraction	Higher energy requirements, cost	^84^
Supercritical CO_2_	High antioxidant levels, established for specialty lipids	Requires cosolvents for polar lipids	^80^
ASE	High recovery rates, efficient separation	Complex setup	^82^
Limonene and ethyl *tert*-butyl ether	Nonhazardous, high selectivity	Higher energy and operating costs	^83,84^

Advancements in solvent engineering have considerably enhanced the selectivity and environmental sustainability of lipid extraction processes. The integration of green chemistry and engineering principles is essential for the long-term viability of microalgal bio-oil production. The selection of renewable and recyclable solvents, the minimization of energy-intensive drying processes, and the valorization of byproducts ought to be prioritized. Life-cycle assessment (LCA) and techno-economic analysis (TEA) allow for the identification of bottlenecks and guidance for process decisions.

## 5 Bio-Oil Extraction: Comparison between Microalgae and Other Oil-Bearing Biomasses

Biofuel production from oilseed biomass has garnered focus due to its potential to replace fossil fuels with renewable source alternatives. Among the various sources, microalgae stand out because of their lipid-rich cell structure, which contrasts sharply with the more complex cell walls of other oilseeds such as soybeans and sunflower seeds. These oilseeds are characterized by higher cellulose and lignin content, which complicate lipid extraction. These structural and compositional characteristics make microalgae a promising option for bio-oil production; however, the ease of cell disruption varies significantly among species, as several commercially relevant strains possess highly robust, multilayered cell walls that may require pretreatment severities comparable to those applied to certain oilseed biomasses.

The comparison between microalgal and terrestrial oil-bearing biomasses is strongly influenced by differences in structural organization and biochemical composition, which determine solvent accessibility, mass transfer resistance, pretreatment severity, and extraction energy demand. Structural factors such as cell wall thickness, lignin content, cellulose crystallinity, and matrix porosity in oilseed crops, as well as the multilayered cell wall structures of certain microalgal species, directly affect solvent diffusion and lipid release mechanisms, thereby influencing the overall extraction efficiency.^10,85^

### 5.1 Cellular Structure and Extraction Efficiency

Microalgae exhibit highly variable cell wall compositions depending on the species: while some strains, such as *Nannochloropsis* and *Phaeodactylum*, have relatively simple membranes, others, such as *Chlorella vulgaris,* exhibit thick, multilayered walls enriched with algae-like polymers or sporopollenin, which directly affect resistance to cell disruption and solvent penetration.^86^ Nonetheless, microalgal biomass generally lacks the dense lignocellulosic matrix found in terrestrial seeds. Oilseeds such as canola, sunflower, and soybean possess rigid cell walls composed of cellulose, hemicellulose, and lignin. The high lignin content and cellulose crystallinity lower the porosity and effective solvent diffusivity, increasing the mass transfer resistance and pretreatment severity. In oilseeds, wall thickness, lignin fraction, and cellulose crystallinity dictate matrix porosity and tortuosity, thereby controlling solvent diffusion and overall mass transfer coefficients. Higher lignin content and crystallinity are typically associated with slower extraction rates and higher required temperatures and residence times.^87^ Walls are lignocellulosic, where cell wall thickness, lignin, hemicellulose, and cellulose crystallinity govern porosity and enzyme/solvent access. Microwave irradiation can increase pore size, pore volume, and surface area while lowering crystallinity, thereby dramatically improving aqueous–enzymatic oil extraction and protein release from peanuts by enhancing enzyme accessibility and mass transfer.^88^

The efficiency of lipid removal is intrinsically associated with the structural complexity of biomass. The cell walls of microalgae, composed primarily of polysaccharides and proteins, are significantly thinner and less complex compared with the lignocellulosic walls of grains and seeds, which are rich in cellulose and lignin.^89^ Shorter diffusion paths and lower lignin content in many microalgae can achieve higher mass transfer coefficients, enabling effective extraction under milder conditions than those used for oilseeds. In species with relatively simple cell wall structures, cell rupture can be achieved with lower pretreatment severity; however, species possessing multilayered or algaenan-rich walls may exhibit recalcitrance comparable to that of some terrestrial oilseed biomasses. Techniques such as ultrasound- and microwave-assisted extraction, which apply rapid pressure and heat, excel in breaking the relatively simple cell walls of microalgae. In contrast, these methods are less effective for lignocellulosic biomass, where the high lignin content poses a significant barrier to accessing the internal lipids. For canola seeds, efficient lipid extraction relies on solvent systems that are often combined with thermal or pressure treatments to penetrate the lignocellulosic matrix. In microalgae, however, solvent diffusion can be enhanced by mild physical pretreatments, reducing solvent usage and preserving lipid integrity.

For oilseeds with lignocellulosic walls, more intensive pretreatment methods are required. Processes like acid or alkaline hydrolysis and high-energy pretreatments, including the use of high-pressure steam, are essential to degrade the cellulose and lignin layers and release the lipids trapped within. For example, efficient lipid extraction from canola seeds hinges on the effective breakdown of lignin, often necessitating the use of potent solvents such as hexane in combination with high-pressure techniques.^90^

In microalgae, cell wall thickness and composition directly determine the breaking force and extraction yield. CRISPR (clustered regularly interspaced short palindromic repeats)-induced thinning of *Nannochloropsis salina* cell walls increased lipid extractability by ∼65% via a greater susceptibility to mechanical stress.^91^ Enzymatic treatments that target cellulose and hemicellulose, such as cellulase, xylanase, pectinase, or combinations like cellulase with laccase, break β-glucosidic bonds and alter the structure of hemicellulose. This results in doubling lipid yields in *Scenedesmus* and approximately a 69% increase in *Nannochloropsis oceanica* when using ethanol, primarily by enhancing solvent access and diffusion pathways.^92,93^

On the other hand, the cell walls of some microalgae species are multilayered and can be more rigid than plant tissues, with trilaminar structures composed of algaenan, cellulose/hemicellulose, and proteins, providing approximately three times the strength of plant cell walls.^85,86^ Species such as *Nannochloropsis*, *Scenedesmus*, and *Chlorella* have high lipid content (30–60 wt %); however, their recalcitrant cell walls hinder solvent diffusion and mass transfer.^10,86^

Robust walls and high water content make drying and mechanical disruption major energy sinks; cell disruption is a key cost bottleneck for algal biodiesel.^10,95,96^ For robust algal walls or biomineralized species, solvent choice can overcome structural limitations, including subcritical or liquefied dimethyl ether. These solvents efficiently extract lipids from wet, “hard-walled” microalgae with minimal or no prior disruption, indicating high solvent penetrability even when conventional solvents are mass transfer limited.^97,98^ In oilseeds, microwave-induced porosity increases enzyme accessibility and hydrolysis rates, directly enhancing the aqueous enzymatic extraction efficiency and lowering the overall energy per unit oil recovered.^88^

Although microalgae present significant advantages for lipid extraction due to their high lipid productivity and potential for controlled cultivation, these benefits are strongly dependent on species selection, cultivation strategy, and the level of process integration adopted in the production system. In contrast, terrestrial oilseed-based production systems currently retain advantages related to technological maturity, an established processing infrastructure, and lower production costs, which continue to support their dominant role in commercial biofuel supply chains.

### 5.2 Advantages of Lipid Extraction from Microalgae over Other Oily Biomasses

Microalgae are known for their high lipid content, which can reach up to 70% of dry biomass depending on the species and cultivation conditions. In contrast, soybeans, sunflowers, and canola present more modest lipid contents, typically ranging from 18% to 45%, as shown in Table 7.

**Table 7:** Comparison of Lipid Content and Productivity of Selected Biomass Feedstocks

Biomass	Lipid Content (% DW)	Oil Yield (L/ha/year)	Productivity (g/m^2^/day)	References
Microalgae	20–70	20,000–80,000	20–50	^99^
Soybean	18–20	∼400–600	∼1.5	^100,101^
Sunflower	35–45	∼800–1,200	∼2.0	
Canola	40–44	∼900–1,200	∼2.2	

Microalgae far exceed terrestrial crops in oil yield per hectare, especially when they are cultivated year-round under optimized conditions. However, commercial-scale productivity remains limited by technological and economic challenges, as mentioned in Section 4.

Extraction methods optimized for microalgae often prove to be ineffective for lignocellulosic biomasses due to their greater structural rigidity. Supercritical CO_2_ extraction, highly efficient for microalgae thanks to their low cellular complexity, encounters significant obstacles with lignocellulosic materials.^63^ The lignin-rich composition of these biomasses demands substantially higher pressures and temperatures to access the internal lipids, making the process more resource intensive.

Similarly, techniques such as ultrasound- and microwave-assisted extraction, which excel in rapidly and efficiently disrupting microalgal cells, are less effective in lignocellulosic seeds. The robust bonds between cellulose and lignin in these biomasses require significantly more energy to break, which reduces the efficiency of these methods. Recent advancements suggest that combining microwave technology with specific solvents could enhance lipid extraction from lignocellulosic biomasses. However, these approaches remain more costly and complex compared to their application in microalgae, where the simple cell structure allows for more straightforward processing. These differences highlight the continued need for tailored strategies to address the challenges posed by lignocellulosic materials.

Microalgae present a compelling alternative to traditional biomasses for biofuel production, offering numerous advantages that position them as a leading candidate for sustainable energy solutions. With exceptional photosynthetic efficiency, microalgae can sequester up to 1.8 tons of CO_2_ per ton of dry biomass, significantly surpassing the capabilities of trees and oilseeds. David et al.^7^ reinforce that microalgal cultivation systems have been reported to fix approximately 1.5–2.0 kg of CO_2_ per kilogram of dry biomass, depending on species productivity, cultivation configuration, and operational conditions. Under favorable irradiation and CO_2_ supply, open raceway ponds typically capture on the order of 20–50 g CO_2_/m^2^/day, while well-optimized photobioreactors can reach approximately 80–100 g CO_2_/m^2^/day due to improved gas transfer and light utilization efficiency. When full system emissions associated with cultivation, harvesting, drying, and conversion are considered, net carbon removal efficiencies generally range from 30 to 70%, depending on process integration, energy sources, and the use of industrial CO_2_ streams or nutrient recycling strategies. In comparison, trees capture approximately 0.5 to 1 ton of CO_2_ per ton of biomass over several years, while oilseeds achieve even lower rates due to their slower growth and lower densities. Moreover, microalgae thrive in controlled environments and can utilize industrial waste streams, such as flue gases, to enhance CO_2_ capture rates. Under optimal conditions, microalgae achieve capture efficiencies of 80–99% when exposed to concentrated CO_2_ sources, such as industrial emissions.^102,103^ Beyond integrating carbon capture, microalgae exhibit unmatched lipid productivity, producing up to 20 times more oil per unit area^104^ than oilseeds like soybeans or sunflowers, a critical factor for industrial scalability. Unlike traditional crops, which require fertile agricultural land, microalgae can grow in wastewater, saline water, and even indoor systems, avoiding competition with food production and enhancing sustainability. Their rapid growth cycle, harvesting in days or weeks as opposed to months for oilseed crops, enables continuous and effective production to meet the steady demand for biofuels.

In addition to bio-oil extraction, microalgae support biorefinery integration, enabling the conversion of residual biomass into biofertilizers, animal feed supplements, and biomaterials. This contrasts with lignocellulosic biomass, which requires complex breakdown processes and lacks the same versatility in downstream applications.

Despite these advantages, large-scale adoption of microalgae faces challenges, including the need for specialized cultivation systems and the high costs of advanced extraction methods such as supercritical CO_2_ extraction. However, ongoing advancements in energy-efficient technologies and hybrid extraction methods are steadily reducing these barriers. Innovations like green solvents and hybrid systems offer promising solutions to address costs and commercial viability, further reinforcing the potential of microalgae as a cornerstone of renewable biofuel production.

The key difference between microalgae and oilseeds is that microalgae biomass is often treated as a true multiproduct biorefinery feedstock, so extraction is designed to preserve proteins and carbohydrates for valorization rather than being optimized solely for oil recovery. Microalgae typically contain 30–60% protein, substantial carbohydrates, and lipids, and economic analyses indicate that viability hinges on valorizing all major fractions in an integrated biorefinery. Because these coproducts are food/feed or fine-chemical grade (e.g., proteins, pigments, polysaccharides), nontoxic, food grade solvents and/or aqueous and enzymatic routes are favored to avoid cross-contamination and reduce downstream purification loads.^105,106^ In contrast, oilseed biorefineries are historically lipid-centric: hexane or similar solvents can be used aggressively because proteins and hull carbohydrates are often downgraded to low-value meal or combustion, easing purity constraints and lowering the per-liter solvent cost and complexity.

For microalgae, downstream processing (harvesting + disruption + extraction + fractionation) can account for 50–60% of total costs in multiproduct concepts; harvesting alone can be 20–40%.^105^ This drives process sequences into the following steps: (1) mild cell disruption; (2) aqueous/enzymatic extraction; and (3) selective lipid separation, which balances some lipid yield for increased total product value and reduced purification energy. Residual protein/carbohydrate-rich algal biomass is often routed to anaerobic digestion, biohydrogen, VFAs, or animal feed, improving carbon utilization and spreading capital across multiple product trains.^106,107^ This integrated routing feeds back into extraction design, favoring methods that leave residues suitable for these uses.

Oilseed systems, with larger, drier seeds and established markets for defatted meal, tolerate harsher thermal and solvent conditions, which simplify lipid extraction even if they partially denature proteins; the economic penalty is smaller than that in high-value microalgal biorefineries. Because microalgal biorefineries depend on monetizing proteins and carbohydrates alongside lipids, they bias toward gentler, often aqueous or food grade extraction schemes that ease purification and enable multiple product chains, even at the cost of more complex fractionation. Oilseed systems, being oil-centric with mature low-value outlets for residues, can prioritize maximal lipid extraction with cheaper, harsher solvents with less stringent integration across fractions.

## 6 Marketing Potential for Bio-Oil Extraction from Microalgae

The commercialization of microalgae bio-oil extraction is gaining momentum, driven by advancements in technologies that improve efficiency and reduce costs. This market holds significant promise because of the unique advantages of microalgae as a renewable high-yield lipid source. The marketing potential of microalgal bio-oil is intrinsically linked to its techno-economic feasibility, quality profile, regulatory context, and compatibility with existing infrastructure. These lipids can be easily converted to bio-oil, with applications spanning the fuel and chemical industries. Increasingly, companies are forming strategic partnerships to develop innovative technologies for microalgae oil production, aiming to transform it into sustainable fuels, such as jet fuel. This trend underscores the growing commercial viability of microalgae-derived products.^108,109^

Reported cost estimates for microalgal bio-oil production vary widely across studies because of differences in system boundaries, cost assumptions, and process integration levels. In some analyses, costs refer only to cultivation or extraction, whereas others include the full production chain, encompassing cultivation, harvesting, dewatering, lipid extraction, and upgrading. Consequently, the ranges presented in this section should be interpreted as indicative values derived from studies using different technoeconomic assumptions rather than as directly comparable production costs.

Despite the growth in the global algal cultivation capacity, current production volumes remain relatively limited. On the basis of leading producing countries, global algal cultivation is estimated to reach approximately 38 million liters of culture volume annually, concentrated mainly in China, Japan, Taiwan, the United States, and India.^110,111^ However, this value refers to the cultivation volume rather than dry biomass output, which varies significantly depending on species, productivity, and harvesting efficiency. The cost of producing microalgae bio-oil can vary greatly over the countries. Figure 3 shows that the prediction cost of microalgae bio-oil is expressed differently in accordance with the specific country’s current basis. Costs can range from USD 0.14 to USD 24.33 per liter, depending on the microalgae strains, production methods, and optimization strategies used.^112^ Another study found the cost to be between USD 0.85 and USD 3.14 per pound, with an average of USD 1.61 per pound when considering byproduct credits.^113,114^

**Figure 3:**
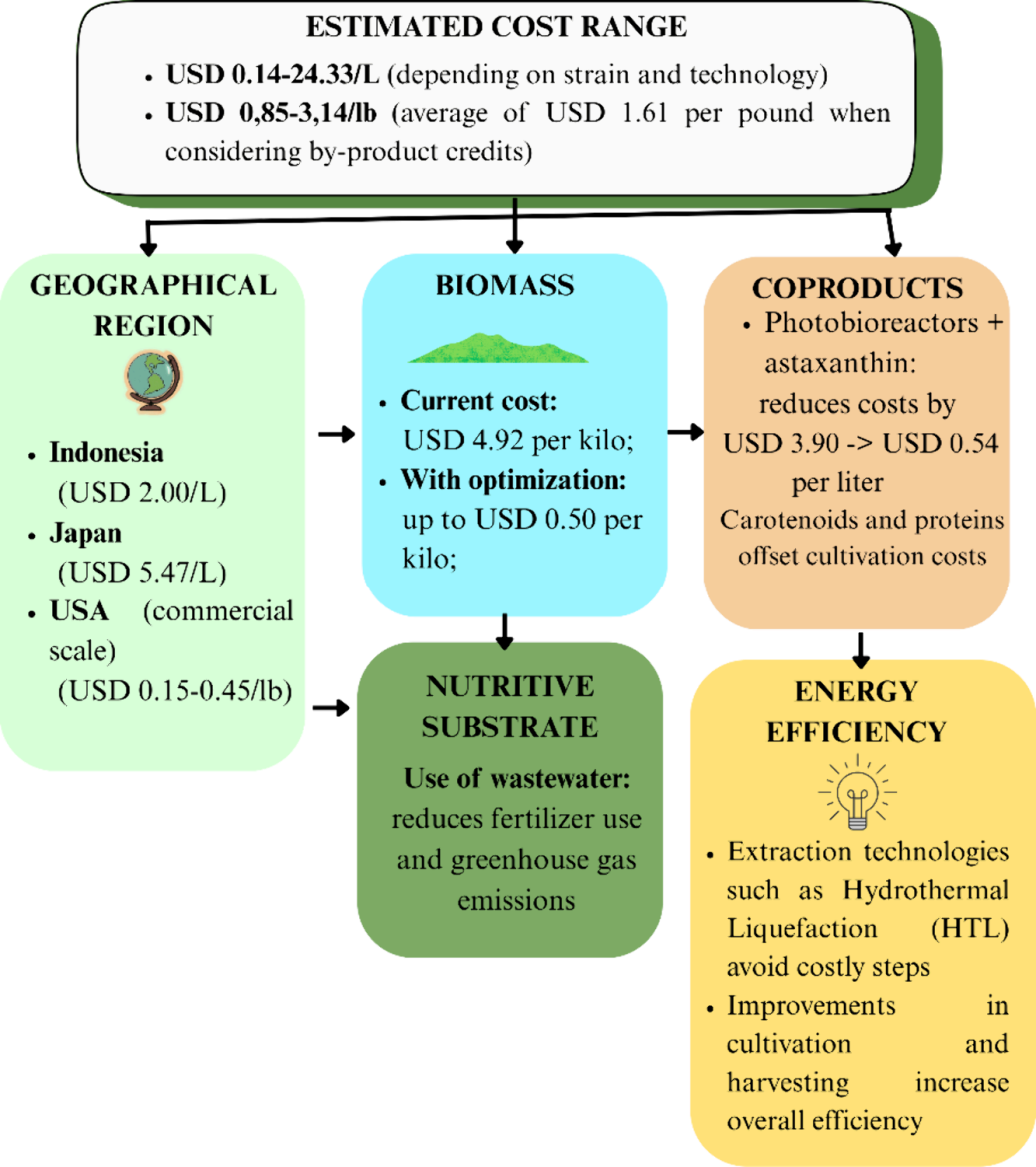
Cost estimates in the process of obtaining bio-oil from microalgae.

In Indonesia, the cost of crude oil from polycultured native microalgae was estimated at USD 2 per liter, while in Japan it was USD 5.47 per liter.^12^ A study conducted on a commercial-scale microalgae farm in the US reported costs ranging from USD 0.15 to USD 0.45 per pound, with an average of USD 0.25 per pound when using improved technology and byproduct credits.^113,114^

The cost of growing and harvesting microalgae biomass is a significant factor. Current technologies result in high expenses, approximately USD 4.92 per kilogram, which is not viable for biodiesel production without substantial advances. However, with process modifications such as HTL, this cost could potentially be reduced to USD 0.50 per kilogram.^13^

Wastewater usage as a nutrient source for microalgae can provide economic and environmental advantages, such as decreasing the dependence on external fertilizers and improving the greenhouse gas balance.^13,115^ High cultivation and harvesting costs, along with the energy-intensive nature of oil extraction, continue to be significant challenges. Strategies to enhance energy efficiency and resource utilization are essential for lowering the overall production costs.

A key development in the bioenergy sector is the integration of extraction technologies with purification processes and coproduct valorization. For instance, combining bio-oil production with the extraction of food grade proteins from microalgae has emerged as an economically viable approach, significantly reducing the overall costs of biorefinery. Optimized cultivation conditions further enhance the competitiveness of microalgae biomass, enabling its utilization across diverse markets including fuels, food, and pharmaceuticals. This multifaceted approach maximizes the value of biomass, contributing to both financial and environmental sustainability.^116^

Microalgae also offer the advantage of adaptability to challenging environments such as saline or wastewater. This capability minimizes the pressure on arable land, providing a sustainable alternative to traditional biofuel feedstocks. However, challenges persist, particularly in terms of their high production costs and energy demands. Innovations in hybrid extraction methods and advances in competitive microalgae-based biofuel production are needed.^117^

The integration of biorefinery processes to maximize the utilization of microalgae biomass offers a sustainable and cost-effective pathway for biodiesel production alongside value-added products. In this approach, the entire biomass is processed to extract a range of components, including lipids for bio-oil, proteins for animal feed, and carbohydrates for bioethanol or specialty chemicals. This “integrated biorefinery” concept ensures that every part of the biomass is utilized, reducing waste and enhancing the economic efficiency of the production process.^109^

By enabling the coproduction of bio-oil and other valuable products such as biofertilizers and additives for the food and cosmetics industries, integrated biorefineries provide a competitive edge. This model not only maximizes economic returns but also promotes sustainability by efficiently repurposing byproducts.^116,118^

Cost reductions can be achieved by cultivating microalgae in nutrient-rich environments, such as wastewater, which transforms waste into a valuable resource. Additionally, coproduction systems that integrate advanced processes, such as fermentation, extraction with green solvents, and the reuse of CO_2_ for cultivating new microalgae, align seamlessly with circular economy principles. These innovations make large-scale production economically viable by ensuring the full utilization of biomass.^119^

Integrating biorefineries with cultivation systems that use industrial CO_2_ emissions and wastewater offers substantial environmental and economic benefits. This approach minimizes environmental impacts, reduces production costs, and establishes a closed-loop system that converts waste into resources, decreasing the dependence on external inputs. When combined with renewable energy sources, this strategy has the potential to achieve economically viable bio-oil production while delivering significant reductions in greenhouse gas emissions.^118^

In addition to production costs, the market potential of microalgae-derived bio-oil is strongly influenced by regulatory and market-side factors, including compliance with fuel quality standards, certification requirements, drop-in compatibility with existing fuel infrastructure, and the availability of policy incentives supporting low-carbon fuels. Furthermore, the competitiveness of microalgal fuels must be evaluated in the context of other advanced biofuel pathways, such as lignocellulosic biofuels and power-to-liquid fuels, which compete for the same policy support mechanisms and market segments. These factors play a decisive role in determining the pace of commercialization and the large-scale deployment of microalgae-based fuel technologies.

Overall, the economic outlook for microalgae-derived bio-oil is shaped primarily by key cost drivers, including biomass productivity, harvesting and dewatering requirements, and downstream processing energy demands. Promising cost reduction opportunities include strain improvement, process integration within multiproduct biorefineries, and the use of waste CO_2_ and nutrient streams. In the near term, commercialization is expected to be more viable in niche markets such as sustainable aviation fuels and specialty biobased products, where higher product values can offset production costs. Nevertheless, uncertainties about large-scale cultivation performance, long-term operational costs, and policy stability remain as significant factors influencing future market deployment.

## 7 Production of E-Fuels from Microalgae Bio-Oil

In the literature, e-fuels and biofuels derived from microalgae are defined and classified. The specific role of microalgae bio-oil within the e-fuel framework is not clearly explained. E-fuels are synthesized using carbon dioxide captured from the environment and hydrogen derived from water electrolysis. These fuels present a promising solution for decarbonizing sectors reliant on internal combustion engines, such as road transportation, maritime shipping, and aviation. This is particularly relevant in regions where the full adoption of electric vehicles faces significant technical and economic hurdles.^120–123^

This section also clarifies that, while biofuels and e-fuels differ in origin (biogenic versus synthetic), they can be synergistically connected within circular carbon systems. E-fuels, or electrofuels, are explicitly defined as synthetic fuels generated by combining CO_2_ (captured from industrial sources, direct air capture, or biogenic processes) with hydrogen produced through water electrolysis powered by renewable energy. The main synthesis routes are Fischer–Tropsch (FT) and methanol synthesis, both of which convert these feedstocks into liquid hydrocarbons or alcohols suitable for use as transportation fuels.^124,125^ Microalgae bio-oils are described as renewable, carbon-rich feedstocks that can be integrated into e-fuel production chains through upgrading processes (e.g., hydrogenation, hydrodeoxygenation, or cofeeding in Fischer–Tropsch synthesis).^126^

To enhance conceptual clarity, an integrative flow diagram (Figure 4) is included to summarize the relationships among the main extraction routes, key performance indicators (yield, energy demand, and environmental impact), and their potential integration with renewable and e-fuel production systems. This framework provides a unified view of material and energy flows across the microalgae-to-fuel value chain.

**Figure 4:**
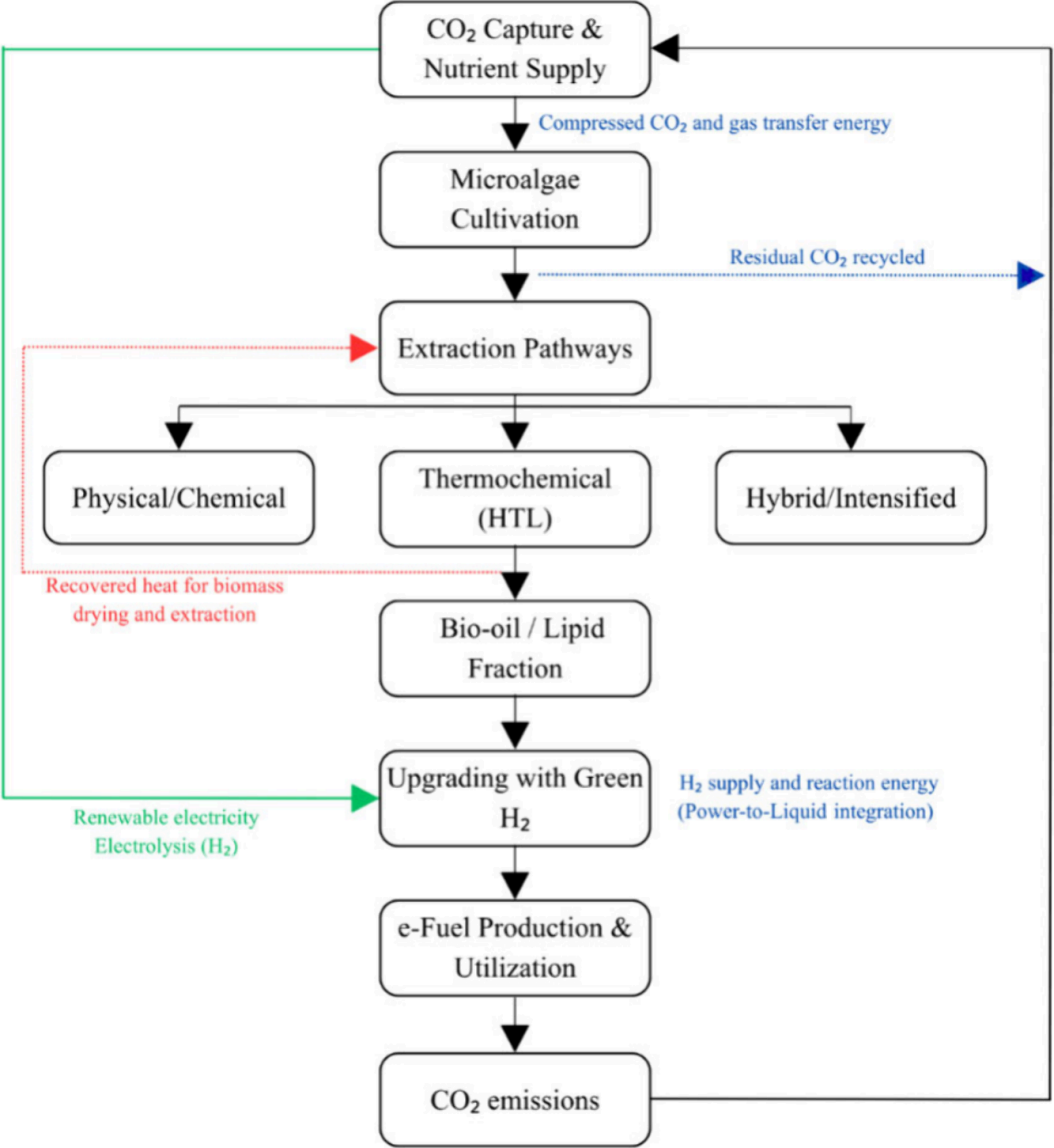
Integrative flowchart: link between CO_2_ capture, microalgae cultivation, extraction routes, upgrading with renewable H_2_, and e-fuel production.

The integrated production cycle for obtaining biofuels and e-fuels from microalgae begins with the supply of CO_2_ (from industrial or biogenic sources) and the use of nutrient-rich streams such as treated effluents. This symbiosis closes the nutrient cycle and reduces the carbon footprint of the system, promoting the continuous supply of carbon and nutrients necessary for intensive microalgal cultivation. This is a central strategy for the sustainability of the microalgal process, according to recent studies.^127,128^

In cultivation, photosynthesis converts CO_2_ into wet biomass rich in lipids, proteins, and carbohydrates. Many industrial processes prioritize the use of wet biomass (20–30% solids), which enables processes such as hydrothermal liquefaction (HTL) and reduces drying energy costs. The integration of heat flows and nutrient recirculation has been identified as key to energy efficiency and economic viability at scale, as demonstrated in continuous-scale experiments with significant yields in the HTL process.^128^

Pretreatment steps such as micro-mechanicalization, ultrasound, enzymatic treatment, or partial drying are routinely employed to increase lipid availability and optimize mass transfer. In the context of e-fuel production, these pretreatments are particularly relevant because they influence the chemical composition of the resulting intermediates, including the concentrations of oxygen- and nitrogen-containing compounds, which affect hydrogen consumption, upgrading severity, and catalyst stability during downstream refining processes.^129,130^

The central block of the flow sheet comprises physicochemical extraction and thermochemical conversion routes that determine the quality of the carbon feedstock entering e-fuel upgrading pathways. Solvent extraction, supercritical CO_2_, and green solvents such as ethanol or ionic liquids can improve selectivity and reduce the coextraction of unwanted components, thereby facilitating subsequent purification and upgrading. Thermochemical conversion by HTL enables the efficient conversion of wet biomass into biocrude; however, the resulting product typically contains significant oxygen and nitrogen fractions, requiring hydrotreatment or catalytic upgrading before integration into synthetic fuel production chains.^23^

Hybrid and integrated processing routes, such as ultrasound-assisted solvent extraction, microwave-assisted pretreatment followed by supercritical CO_2_ extraction, or catalytic HTL, are increasingly explored to improve carbon recovery while simultaneously reducing heteroatom content and enhancing compatibility with downstream upgrading and e-fuel synthesis processes. Recent pilot-scale studies indicate that such integrated approaches can improve energy efficiency while producing intermediates more suitable for hydrogenation and thermochemical fuel upgrading.^23,130^

Advanced pyrolysis is an emerging approach for converting microalgae biomass to bio-oil with improved energy efficiency. Catalytic pyrolysis, in particular, has been studied to enhance bio-oil yield and quality by minimizing undesirable compounds such as oxygenates and nitrogenous components. This refinement increases the energy density of the final product, making pyrolysis a promising technique for large-scale applications.^78,131^

The products of the extraction and conversion stages, lipid fractions and biocrude, require subsequent upgrading, usually via hydrogenation, hydrodeoxygenation, and hydrocracking, processes that consume large volumes of H_2_. Therefore, access to renewable H_2_, derived from electrolysis powered by renewable energy, is a critical requirement to ensure a low carbon intensity of the entire chain. Removing nitrogen and sulfur contaminants is essential to avoid catalyst poisoning, a recurring focus in detailed technical studies.^132^

The hydrogen demand associated with upgrading processes is substantial, typically ranging from approximately 20 to 60 g of H_2_ per kilogram of biocrude, depending on the feedstock composition, heteroatom content, and upgrading severity. Continuous hydrotreating systems targeting near-complete deoxygenation and denitrogenation typically operate within the range of 35–45 g H_2_ kg^–1^, highlighting the importance of integrating renewable hydrogen production and process heat recovery to maintain favorable overall energy balances.^133,134^

In the synthesis of e-fuels, two main routes stand out: the generation of syngas from bio-oil (via reforming/activation) followed by Fischer–Tropsch synthesis, or the direct conversion of CO_2_ + H_2_ into high-value hydrocarbons (power-to-liquid). Integrating microalgal production with renewable carbon supplies favors a positive life-cycle balance, provided that (i) the H_2_ is from a renewable source and (ii) the logistics and energy balance of the process are properly optimized. Recent reviews emphasize that the reuse of heat and coproducts is essential for systemic sustainability.

This integration not only links microalgal bio-oil extraction to e-fuel synthesis but also enables energy recovery and carbon recycling within a closed-loop system. Heat from hydrothermal or catalytic upgrading can be reused for biomass drying or extraction, and residual CO_2_ from the upgrading stage can be redirected to the cultivation system. Such synergies enhance the overall energy efficiency and carbon utilization, as supported by recent techno-economic and modeling studies. Comparable system integration benefits have been highlighted in recent techno-economic and process integration analyses, which identify heat recovery, hydrogen integration, and carbon recycling as key drivers of improved overall performance for integrated microalgae-to-fuel pathways.^6,17^

Energy integration pathways are also shown in Figure 4 (dashed lines), including heat recovery from upgrading to extraction and drying, reuse of biogas for process heat, and coupling of renewable electricity to CO_2_ capture and hydrogen production. These flows improve the overall energy efficiency and enable partial self-sufficiency of the integrated system.

Although microalgal bio-oils do not fit the strict definition of e-fuels, they remain important in renewable fuel strategies as a renewable, carbon-rich feedstock that can be upgraded and coprocessed with fossil or synthetic intermediates to produce replacement fuels.^135^ Both e-fuels and advanced biofuels, including those from algae, offer pathways to reducing greenhouse gas emissions; nonetheless, they face challenges such as high production costs, scalability, and the need for significant investment and policy support.^136^

E-fuels are currently more expensive than fossil fuels, with mitigation costs estimated at between EUR 800 and EUR 1,200 per ton of CO_2_, although large-scale deployment and technological advances could reduce these costs over time.^123^ Therefore, process intensification, catalyst innovation, and improved reactor designs can achieve the required efficiency and economic viability for e-fuel production.

Market projections suggest that e-fuels could reach a 10% share within five years and up to 30% within two decades, especially in transport and industry; however, this will require overcoming current economic and technical barriers.^137^ A diversified approach that includes both e-fuels and renewable bio-oils, such as those from microalgae, is likely needed to achieve more efficient decarbonization that is meaningful for the energy sector.^123^

The pressing need to reduce greenhouse gas (GHG) emissions is underscored by the alarming increase in global emissions, which surpassed 53.8 billion tons in 2023, an increase of 13 billion tons since 2000.^138,139^ This escalation has directly contributed to rising global average temperatures, exacerbating environmental degradation, intensifying natural disasters, and causing substantial economic loss. Consequently, the urgent reduction of carbon dioxide emissions has become imperative with the transition from fossil fuels to cleaner alternatives identified as a critical strategy.

Nonfossil synthetic fuels, including biofuels and e-fuels, emerge as key solutions. Biofuels harness photosynthesis to convert solar energy into chemical energy through biomass, while e-fuels are produced by reacting hydrogen (H_2_) with captured carbon sources, such as carbon dioxide (CO_2_) or carbon monoxide (CO). The resulting liquid or gaseous fuels possess properties similar to those of traditional fossil fuels, enabling their use in conventional engines without any significant modifications. Moreover, e-fuel production can seamlessly integrate into existing fuel distribution and storage infrastructures, lowering barriers to large-scale adoption.^138,140,141^

A notable example of e-fuel development is Porsche’s investment in a production facility in Chile, leveraging wind energy to synthesize e-fuels. This initiative demonstrates the feasibility of producing e-fuels in regions with abundant renewable energy resources.^142^

On the other hand, advancements in CO_2_ capture technologies and the utilization of renewable energy remain critical, and e-fuels offer a valuable complementary solution in the energy transition. They are particularly suited for sectors that are difficult to electrify and can be used within existing transport infrastructure and vehicle fleets, thereby contributing to a diversified approach to decarbonization.^143^

The synergy between e-fuels and microalgae presents an innovative avenue for sustainable energy solutions. Microalgae are highly efficient biomass sources, characterized by rapid growth rates and a high lipid content. In addition, they capture carbon dioxide during photosynthesis, further reducing emissions.

Bio-oil derived from microalgae serves as a versatile precursor for e-fuels, either through direct conversion to biodiesel or as a feedstock for chemical synthesis. This conversion can be achieved via two primary methods: hydrogenation, where bio-oil is refined to produce synthetic hydrocarbons, and the Fischer–Tropsch process, where CO_2_ and H_2_ are synthesized into liquid fuels using microalgae bio-oil as a carbon source.

Microalgae contribute to carbon sequestration, acting as natural sinks that reduce the level of CO_2_ emissions across the e-fuel life cycle. Although the high costs and large-scale challenges remain significant barriers, advancements in cultivation techniques and integrated biorefineries offer promising routes for improving the efficiency and economic feasibility. Notable developments include genetic engineering to enhance lipid productivity and energy conversion yields, as highlighted in recent studies that have been analyzed and discussed.

This technological convergence positions microalgae as an innovative solution for hard-to-electrify sectors such as aviation and maritime transport. Nevertheless, continued research and development are essential to overcoming economic and technical barriers, facilitating large-scale implementation, and reducing environmental impacts.

### 7.1 Hydrogenation

Bio-oil hydrogenation represents a promising technological route for producing synthetic hydrocarbons from renewable resources. Bio-oil, from lignocellulosic biomass or microalgae, contains a complex mixture of oxygenated compounds including carboxylic acids, phenols, aldehydes, and ketones. However, its high chemical instability and elevated oxygen content limit its direct use as fuel or industrial feedstock.^144^ Hydrogenation treatment is therefore critical to reduce the oxygen content, enhancing the stability and calorific value of bio-oil. Figure 5 illustrates a simplified mechanism of the bio-oil hydrogenation reaction.

**Figure 5:**
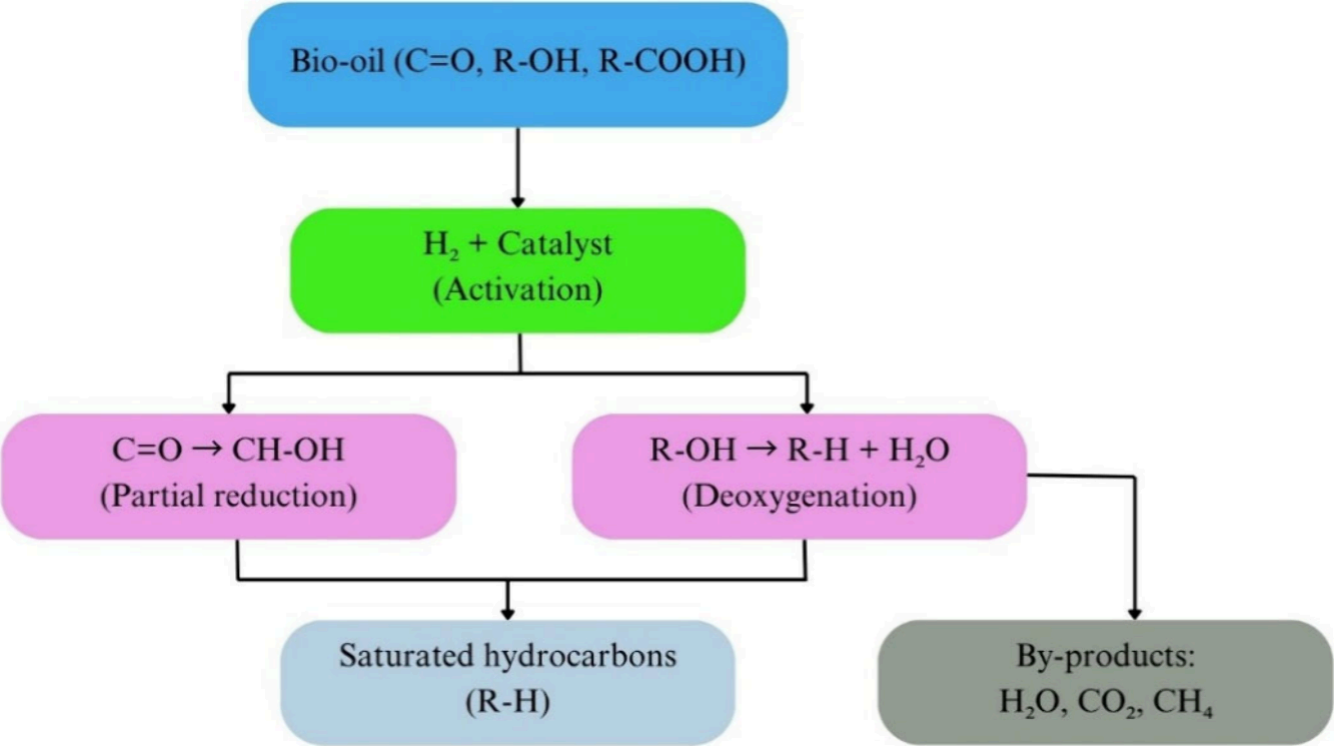
Reaction mechanism in bio-oil hydrogenation.

Microalgae-derived biocrudes typically exhibit higher nitrogen and oxygen content, as well as inorganic impurities and ash-forming species, which can accelerate catalyst deactivation and increase hydrogen consumption during upgrading. These characteristics require careful catalyst selection, pretreatment, or guard-bed strategies for contaminant removal and optimization of upgrading severity to balance fuel quality improvement with catalyst lifetime and process economics.^134,145,146^

The hydrogenation process can be divided into three main stages: pretreatment of the bio-oil, the hydrogenation reaction itself, and the refining of the resulting products. During pretreatment, the bio-oil is separated into fractions via distillation to remove contaminants such as heavy metals and ash, which can deactivate catalysts.^147^ In some approaches, mixing bio-oil with solvents such as water or hydrocarbons improves reactivity during the process.

The hydrogenation reaction occurs under high-temperature (200–400 °C) and high-pressure (50–200 bar) conditions in the presence of catalysts. Commonly used catalysts include metals such as nickel (Ni), cobalt (Co), and molybdenum (Mo) supported on alumina (Al_2_O_3_), as well as noble metals like platinum (Pt) and palladium (Pd). These catalysts facilitate the activation of molecular hydrogen and the removal of oxygen as water vapor. During the reaction, oxygenated compounds adsorb onto the catalyst, and activated hydrogen breaks C–O bonds and saturates double bonds, producing stable hydrocarbons.^147,148^

Postreaction, the products are refined through separation by distillation to yield specific fuel fractions, such as gasoline, diesel, and kerosene.^149^ Gaseous byproducts like methane (CH_4_) and carbon dioxide (CO_2_) can be recycled within the process or converted into energy.

Despite its potential, bio-oil hydrogenation faces technical challenges, including catalyst deactivation due to coke formation and poisoning by sulfur- and nitrogen-containing compounds. High hydrogen consumption is another limitation that can be addressed by using green hydrogen produced from renewable sources. The integration of hydrogenation processes into biorefineries and the development of more efficient and durable catalysts are critical strategies for enhancing the economic viability and environmental sustainability.^150,151^

### 7.2 Fischer–Tropsch Processes

The Fischer–Tropsch process is a catalytic reaction that converts synthesis gas, a mixture of carbon monoxide (CO) and hydrogen (H_2_), into liquid hydrocarbons. When carbon dioxide (CO_2_) is used as the carbon source, the process is adapted to include the conversion of CO_2_ to CO via the reverse water–gas shift (RWGS) reaction before the Fischer–Tropsch step.^152^ Figure 6 depicts a simplified reaction mechanism of the Fischer–Tropsch process.

**Figure 6:**
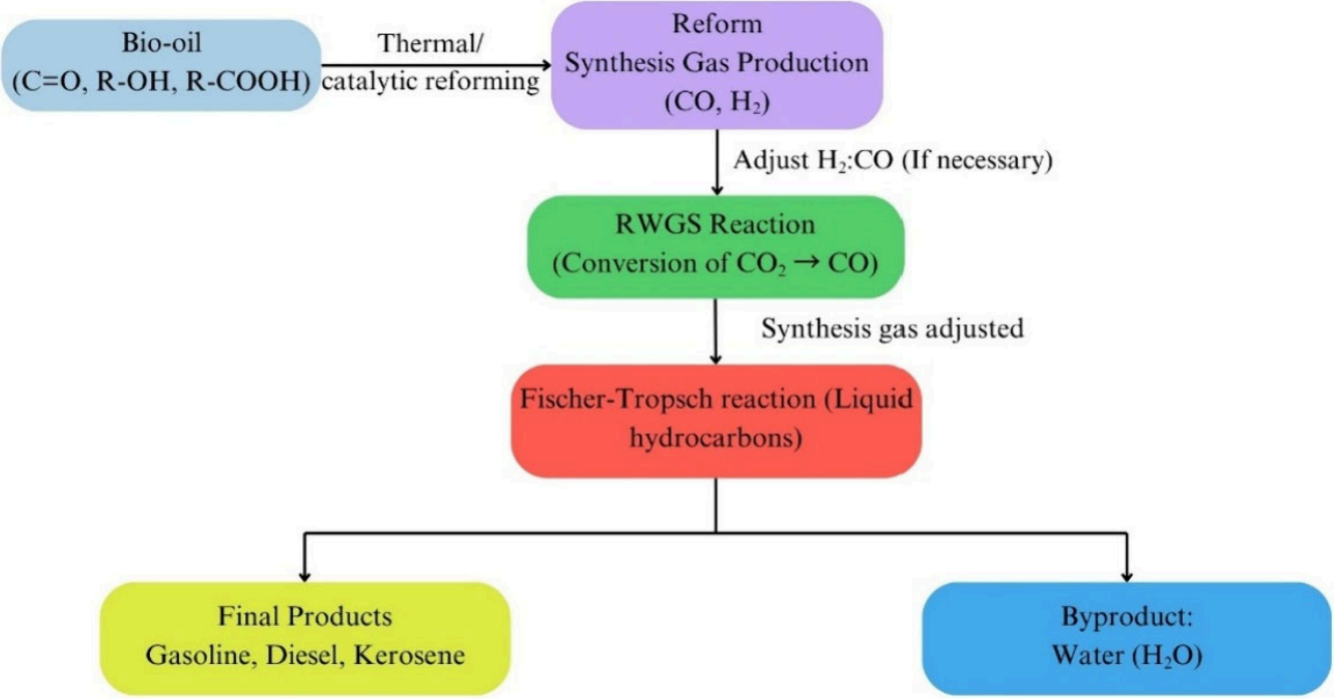
Fischer–Tropsch reaction mechanism from bio-oil.

When microalgal bio-oil reformate is used as a syngas source, advantages include the utilization of renewable biogenic carbon and compatibility with integrated biorefinery schemes. However, compared with direct CO_2_-to-syngas routes, reformate-derived syngas may contain impurities requiring additional gas-cleaning steps, and carbon efficiency can be influenced by reforming losses and upstream upgrading requirements. Conversely, CO_2_ hydrogenation routes benefit from simpler feedstock purity control but depend heavily on the availability of low-cost renewable hydrogen, highlighting the trade-offs among carbon utilization efficiency, impurity management, and overall process complexity.^153–155^

Bio-oil extracted from microalgae can serve as a carbon source for this process because of its oxygen- and carbon-rich composition, which includes fatty acids and other organic compounds. Through thermal, catalytic, or thermochemical reforming, bio-oil can be converted into synthesis gas containing CO and H_2_, which is then fed into the Fischer–Tropsch synthesis.

The initial stage involves the production of synthesis gas via thermal or catalytic reforming methods, such as steam reforming or partial oxidation, to generate H_2_ and CO. If the synthesis gas has a high proportion of CO_2_, the RWGS reaction is used to adjust the gas composition:

$${\mathrm{CO}}_{2}(g)+H_{2}(g)↔\mathrm{CO}(g)+H_{2}O(g)$$ 1

The adjusted synthesis gas is then introduced into the Fischer–Tropsch reactor, which converts it into liquid hydrocarbons using iron (Fe) or cobalt (Co)-based catalysts. The reaction occurs at moderate temperatures (200–350 °C) and high pressures (10–40 bar), with precise control of the H_2_:CO ratio. The resulting products include paraffins, olefins, and oxygenated compounds, which can be refined to produce fuels such as gasoline, diesel, and kerosene.

## 8 Final Remarks and Future Perspectives

This review critically evaluates bio-oil extraction pathways from microalgae, focusing on their role in enabling sustainable e-fuel production. By comparing hydrothermal liquefaction (HTL), ultrasound- and microwave-assisted extraction, supercritical processes, and green solvent systems, it identified how extraction choices influence the overall process efficiency, energy demand, and compatibility with downstream e-fuel synthesis.

In the short term, HTL emerges as the most promising techno-economic option for wet biomass, primarily because of its ability to process high-moisture feedstocks without energy-intensive drying and its potential for direct integration with upgrading and e-fuel production units. Ultrasound- and microwave-assisted methods, especially when applied in hybrid configurations, can enhance conventional extraction routes by shortening processing times, improving yields, and reducing solvent consumption, thereby supporting niche and multiproduct biorefinery concepts. Although supercritical CO_2_ extraction currently entails higher capital and operating costs, its high lipid recovery, tunable selectivity, and lower environmental footprint position it as a strategic option for future, more-mature deployment scenarios.

From a technological roadmap perspective, the findings of this review highlight that wet biomass streams are better suited to hydrothermal and integrated upgrading routes, whereas dry biomass streams are more compatible with solvent-based extraction followed by conventional refining or catalytic upgrading to e-fuels. Near-term commercialization is most likely to occur in diversified biorefineries that coproduce fuels, chemicals, and high-value coproducts, rather than in large-scale, fuel-only plants. Achieving broader deployment of microalgae-derived e-fuels will require progress in cultivation productivity, low-energy harvesting and dewatering, cost-effective CO_2_ capture, and access to low-carbon hydrogen for upgrading.

The overview analysis shows that no single technology is best for all cases; instead, process design should align with biomass characteristics, regional resources, and project timelines. Future efforts should focus on developing hybrid, intensified extraction–conversion techniques that reduce energy use while also conducting techno-economic and life-cycle assessments that include carbon capture and hydrogen supply. Furthermore, pilot and demonstration projects are essential to validate integrated microalgae-to-e-fuel systems. Developing extraction techniques that meet these system requirements is crucial for microalgae-derived bio-oils to play a significant role in large-scale decarbonization and to support the global energy shift.
